# Exogenous (automatic) attention to emotional stimuli: a review

**DOI:** 10.3758/s13415-014-0270-2

**Published:** 2014-03-29

**Authors:** Luis Carretié

**Affiliations:** Facultad de Psicología, Universidad Autónoma de Madrid, 28049 Madrid, Spain

**Keywords:** Exogenous attention, Automatic attention, Emotion, Preattention, Reorienting, Sensory amplification, ERPs, fMRI

## Abstract

Current knowledge on the architecture of exogenous attention (also called automatic, bottom-up, or stimulus-driven attention, among other terms) has been mainly obtained from studies employing neutral, anodyne stimuli. Since, from an evolutionary perspective, exogenous attention can be understood as an adaptive tool for rapidly detecting salient events, reorienting processing resources to them, and enhancing processing mechanisms, emotional events (which are, by definition, salient for the individual) would seem crucial to a comprehensive understanding of this process. This review, focusing on the visual modality, describes 55 experiments in which both emotional and neutral irrelevant distractors are presented at the same time as ongoing task targets. Qualitative and, when possible, meta-analytic descriptions of results are provided. The most conspicuous result is that, as confirmed by behavioral and/or neural indices, emotional distractors capture exogenous attention to a significantly greater extent than do neutral distractors. The modulatory effects of the nature of distractors capturing attention, of the ongoing task characteristics, and of individual differences, previously proposed as mediating factors, are also described. Additionally, studies reviewed here provide temporal and spatial information—partially absent in traditional cognitive models—on the neural basis of preattention/evaluation, reorienting, and sensory amplification, the main subprocesses involved in exogenous attention. A model integrating these different levels of information is proposed. The present review, which reveals that there are several key issues for which experimental data are surprisingly scarce, confirms the relevance of including emotional distractors in studies on exogenous attention.

## Introduction

A considerable number and variety of hazards and valuable resources, often unexpected, are continuously involved in the life of an organism. The evolutionary response to this permanent pressure has been the development of a wide range of strategies, from physical to cognitive, that enable an appropriate response. At the cognitive level, one key survival tool is the efficient monitoring, detection, and processing of these biologically salient events even when the individual is engaged in a resource-consuming task, so as to cope with them if necessary. This efficiency relies on exogenous attention, also called automatic, stimulus-driven, or bottom-up attention, among several other terms. Indeed, exogenous attention can be understood as an adaptive tool that permits the detection and processing of biologically salient events that appear out of the current focus of attention.

**Fig. 1:**
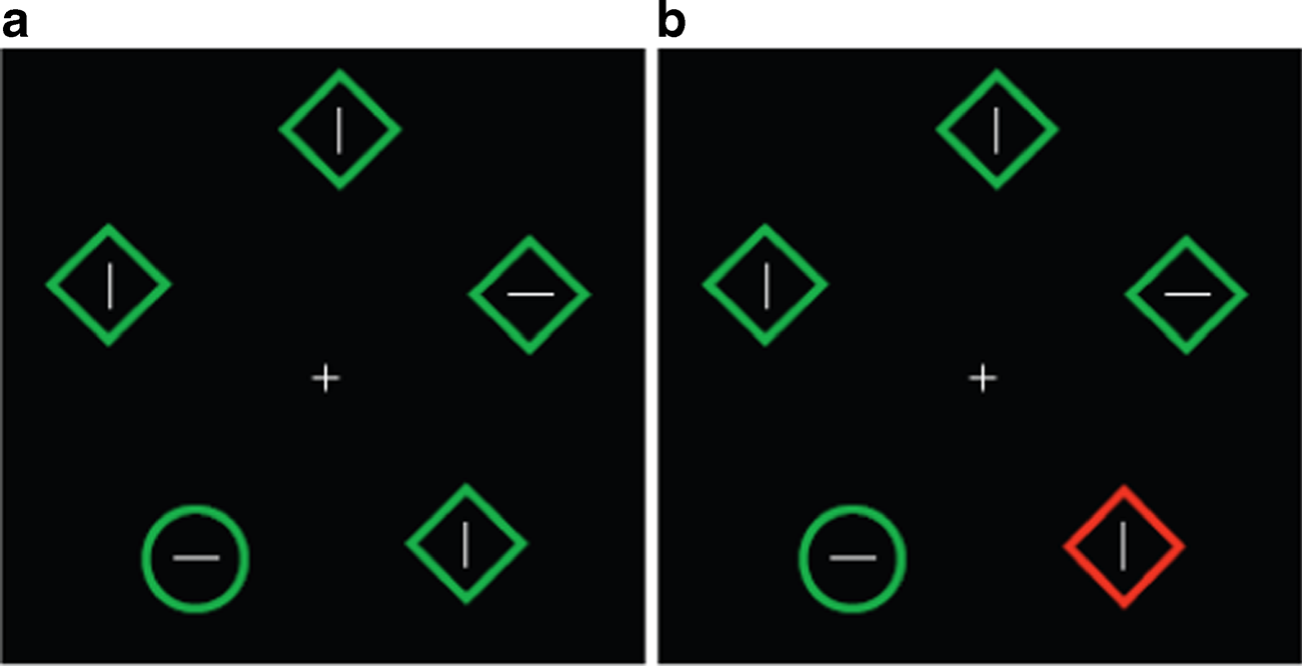
Example of exogenous attention task in which participants are asked to indicate the orientation of the line within the circle. **a** Control condition. **b** One distractor is manipulated to capture attention. Adapted from de Fockert, Rees, Frith and Lavie (2004)

As Yantis (1993) pointed out more than 2 decades ago, exogenous attention has been much less studied than endogenous attention, a bias that has prevailed up to the present. Endogenous attention, also called top-down, voluntary, or controlled attention, is goal-driven and directed toward the events or stimuli consciously decided by the individual to be targets of processing. Exogenous attention could be conceptualized as a sort of interruption of endogenous attention or, more precisely, as a reorientation of endogenous attention to a different stimulus or to a different characteristic of the currently attended stimulus. Indeed, visual tasks exploring exogenous attention typically consist of asking participants to direct their endogenous attention to a particular element (e.g., “the orientation of the line within the green circle”) presented among other, irrelevant, endogenously unattended elements or *distractors* (e.g., green diamonds with their own line inside). In some experimental conditions within the experiment, distractors are manipulated so that they tend to capture attention (e.g., one distractor is drawn in red; see Fig. 1).

Typically, capture of exogenous attention by distractors causes disruption in the ongoing task, which is reflected in poorer processing of targets: Reaction times and/or errors in the task increase (e.g., using the task depicted in Fig. 1; de Fockert et al., 2004; Hickey, McDonald & Theeuwes, 2006; Theeuwes, 1992). These two behavioral signals of attentional capture by distractors are, by far, those most employed in research on exogenous attention, but other indices also exist. One of them is ocular activity: Saccades in the ongoing task are altered to a greater extent by distractors capturing attention than by distractors unable to capture it (McSorley, Cruickshank & Inman, 2009). Reorientation of attention to distractors is also associated with several autonomic changes, such as bradycardia, increase in skin conductance, or mydriasis (Öhman, Esteves, Flykt & Soares, 1993; Sokolov, 1963; Spinks & Siddle, 1983). However, all of these signals, which reliably reveal whether distractors actually capture attention, are insufficient to characterize the underlying mechanisms controlling exogenous attention. Neural information is necessary for these purposes.

### Characterization of exogenous attention to nonemotional stimuli

**Fig. 2:**
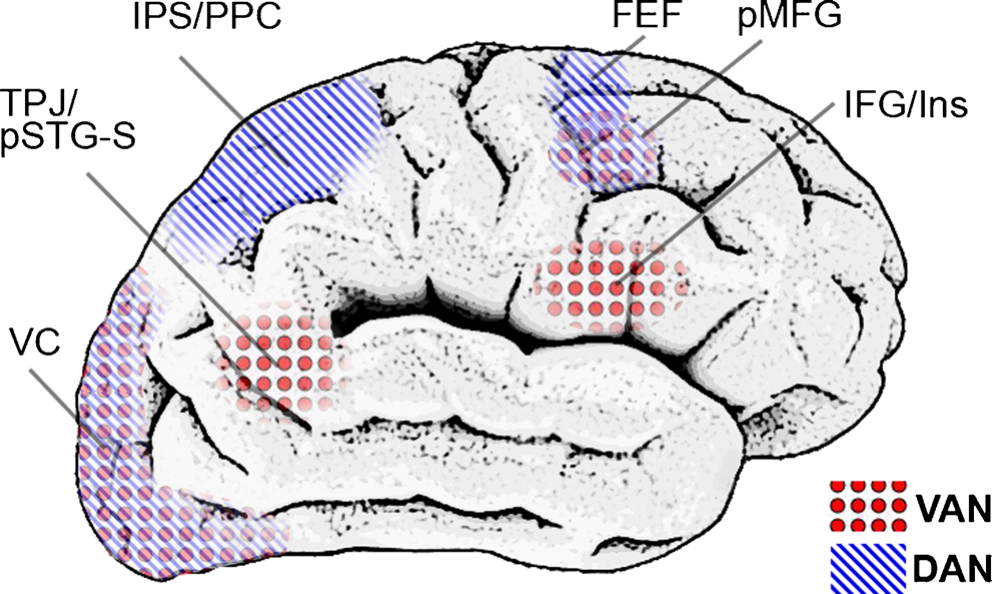
Main cerebral areas involved in exogenous attention to nonemotional stimuli. This schematic (nonexact) anatomical illustration summarizes data provided in different reviews (Corbetta, Patel & Shulman, 2008; Ptak, 2012; Smith & Schenk, 2012). Areas filled with red dots belong to the ventral attention network (VAN), and those filled with blue lines belong to the dorsal attention network (DAN). Please note that some areas, such as the pMFG, have been proposed as belonging to both networks. VC = visual cortex, TPJ = temporo-parietal junction, pSTG-S = posterior part of the superior temporal gyrus-sulcus, IPS = intraparietal sulcus, PPC = posterior parietal cortex, FEF = frontal eye field, pMFG = posterior part of the middle frontal gyrus, IFG = inferior frontal gyrus, Ins = insula

According to different theories and models, exogenous attention involves different processes—preattention, reorienting, and sensory amplification being the most important. The following definitions regard the visual domain, on which the present review will focus. *Preattention* consists of the continuous and automatic monitoring and evaluation of the environment, taking into account also stimuli that project to peripheral, nonfoveal areas of the retina, where perception is poorer (e.g., Jonas, Schneider & Naumann, 1992). In fact, preattention is proposed to be carried out through low-load and fast processing systems that work on low-level stimulus features (Graham, 1997; Öhman, 1979; Theeuwes, 1992). *Reorienting*, or orienting response, is defined as the automatic orientation of processing resources—for example, through gaze or head motion—toward those events considered important by preattention/evaluation structures (Graham & Hackley, 1991; Siddle, Stephenson & Spinks, 1983; Sokolov, 1963), while disengaging from the ongoing task (Corbetta & Shulman, 2002; Posner, Rueda & Kanske, 2007). In the visual domain, orienting response pursues the foveal projection of stimulation. It is important to note, however, that these motor-spatial reorienting mechanisms, although often necessary, are not mandatory in exogenous attention, since sometimes the element or characteristic exogenously capturing attention is located close to, or in, the endogenously attended location. And finally, *enhanced sensory processing* of the important event, which is also within the scope of internally driven, endogenous attention, consists of the modulation of perception-related neural mechanism so that the processing of the stimulus capturing attention is potentiated (Asplund, Todd, Snyder & Marois, 2010; Serences & Yantis, 2007). This heterogeneous set of processes would be supported by different neural networks whose architecture has mainly been characterized through neutral, *nonemotional* stimulation (see Fig. 2).

On the one hand, studies providing spatial information on brain activity have revealed the dorsal and ventral attention networks (DAN and VAN, respectively) as two key circuits underlying different aspects of exogenous attention. Both networks have been comprehensively reviewed elsewhere (e.g., Corbetta et al., 2008; Corbetta & Shulman, 2002). Some of the best defined structures involved in VAN are the temporo-parietal junction and neighboring areas in the posterior part of the superior temporal gyrus and sulcus, which form a tandem with (and are probably controlled by) the lateral-caudal frontal cortex—namely, the posterior areas of the inferior frontal gyrus and the insula. The VAN would be responsible for changing from internally directed processes to environmentally directed processes (Corbetta et al., 2008; Posner et al., 2007). Several dorsal areas, including the superior parietal lobule and dorsal-caudal frontal regions such as the frontal eye fields and motor and premotor areas (mainly in the posterior part of the middle frontal gyrus and in the precentral gyrus), form the DAN and have been linked, respectively, to limb and eye motion planning for coping with the distractor (Heed, Beurze, Toni, Röder et al., 2011) and to eye reorientation itself (Posner et al., 2007), the latter task also involving midbrain and thalamic nuclei (Baker, Patel, Corbetta & Snyder, 2006; Kirchner, Barbeau, Thorpe, Regis et al., 2009). Once processing resources are oriented to the distractor, sensory processing is enhanced in order to facilitate its processing, and consequently, greater activity in visual cortices is observed (Asplund et al., 2010; Rees, Frith & Lavie, 2001; Serences & Yantis, 2007).

On the other hand, data on the timing of the different phases within this process have been also reported. These phases would be reflected in different components of the event-related potentials (ERPs), the neural signal most frequently measured in this area of research. In the specific case of visual stimulation experiments, studies on exogenous attention to nonemotional stimuli have reported effects in three early components: in chronological order from 100 to 250 ms, P1 (see a review in Hopfinger & Mangun, 2001), anterior P2 (Kenemans, Verbaten, Melis & Slangen, 1992; Kenemans, Verbaten, Roelofs & Slangen, 1989), and N2 (see reviews in Folstein & Van Petten, 2008; Pazo-Alvarez, Cadaveira & Amenedo, 2003). Later exogenous attention effects have also been reported for N2pc, a component maximal at parietal areas contralateral to the stimuli capturing attention (Hickey et al., 2006; but see Wykowska & Schubö, 2011), and, consistently, in P3a and other late positivities that often require specific experimental paradigms (e.g., oddball tasks; see reviews in Polich, 2003, 2007). Studies linking these components to structures mentioned above through source localization algorithms are still scarce, but existing data provide relevant information. P1 is mainly elicited in visual cortices (Di Russo, Martínez, Sereno, Pitzalis et al., 2002). Subsequent P2 and N2 have been reported to originate in a variety of areas, including visual cortices and VAN/DAN, among other structures (Carretié, Albert, López-Martín, Hoyos et al., 2013; Carretié, Hinojosa, Mercado & Tapia, 2005; Carretié, Kessel, Carboni, López-Martín et al., 2013; Luck, 1994; Schönwald & Müller, 2014). P3a and other late positivities, which have been proposed to reflect the automatic-controlled frontier since they are strongly modulated by top-down processes, present broad spatial contributions including sensory areas (Polich, 2007; Weinberg, Ferri & Hajcak, 2013). As these observations suggest, and as is discussed in more depth in the Temporal Characterization: Main Phases section, different subprocesses of exogenous attention appear to occur mostly in parallel, rather than in purely serial fashion, with enhanced sensory processing being manifested from early to late latencies at the same time as reorienting mechanisms linked to VAN and DAN.

### The present review

#### Justification and scope

As was indicated, the information described above was obtained in nonemotional tasks (distractors were nonemotional stimuli). These studies have provided extensive and crucial knowledge on exogenous attention and have identified the main elements making up the mechanisms underlying this process. However, taking into account the key role of exogenous attention in detecting biologically salient events, which are usually charged with affective meaning, experiments including emotional distractors are valuable research contributions in this field. From the cognitive science perspective, it is widely acknowledged that the anatomy of exogenous attention has not yet been fully described (Corbetta et al., 2008). While, as we have just seen, the reorientation of processing resources toward the distractor and the sensory enhancement mechanisms are relatively well defined, preattention/evaluation needs further characterization. Up to now, models of exogenous attention have not clearly attributed this function to any particular node of the VAN and DAN, but some clues can be found in other lines of research. As was indicated, evaluative structures would be those responsible for discriminating salient stimuli—usually emotional—from anodyne/neutral stimuli through automatic, low-cost conditions. Although exogenous attention to emotional stimuli is a relatively new area of study (as we are about to see, it is an eminently 21st-century research field), several experiments reviewed here suggest some candidates for preattentional/evaluative brain structures, as will be described later.

From an affective science perspective, disentangling mechanisms responsible for attending to emotional stimuli from other processes, such as those associated with the organization of autonomic or hedonic responses, is a relevant and active line of research (Pourtois, Schettino & Vuilleumier, 2012). An important related question is how an “emotional stimulus” is conceptualized. In this review, a basic, general perspective is adopted: An emotional stimulus is any event capable of triggering emotional reactions, at any level (physiological, subjective, and/or behavioral) and to any extent, in the receiver. Please note, however, that different and more specific definitions have been proposed in affective sciences from diverse theoretical frames (Brosch, Pourtois & Sander, 2010).

The present review is aimed at answering two main, general questions. The first one is whether there is a *quantitative* difference between exogenous attention to nonemotional and to emotional distractors—that is, whether emotional stimuli enhance the intensity of those indices of exogenous attention described above to a greater extent than do nonemotional (or emotionally neutral) stimuli. The second question is of a *qualitative* nature and deals with the mechanisms underlying exogenous attention to emotional stimuli. The answer mainly—but not exclusively—requires cerebral information, which is also reviewed here. The question is whether the well-known neural circuitry described for neutral distractors is also valid for emotional distractors and/or whether it is enriched or complemented by other brain regions in the latter case.

#### Selection of studies

Parallel to the diverse nomenclature with which exogenous attention is labeled (some synonyms were given at the beginning of the Introduction), there is also great diversity regarding the experimental paradigms with which it can be explored. While all of them provide important clues in relation to this process, it is unlikely that any of them inform us about “pure” or “isolated” exogenous attention. Indeed, it is difficult to disentangle from other processes, particularly from those forming the metacategory of “executive processes,” such as task switching, response inhibition, or conflict resolution, which are necessarily present in situations where distractors capture attention, diverting it from the ongoing task. In any case, this review has left out those experiments in which other cognitive processes besides exogenous attention, such as memory, are explicitly demanded in the ongoing task.

**Fig. 3:**
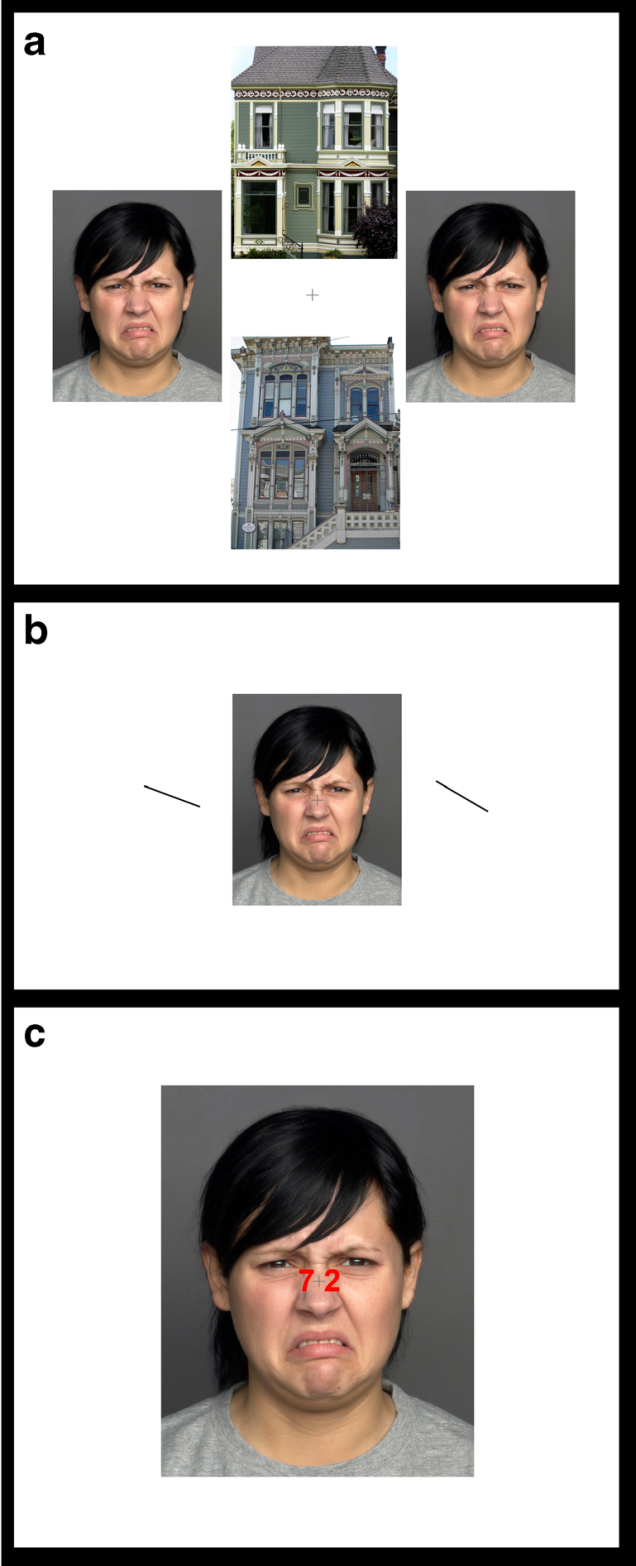
Examples of some frequently employed concurrent but distinct target–distractor tasks using the same distractor (obtained from FACES database; Ebner, Riediger & Lindenberger, 2010; http://faces.mpib-berlin.mpg.de). **a** Face–house task: The relevant instruction regarding exogenous attention to emotional distractors asks participants to indicate whether houses (target) are the same or different within each trial. **b** Line orientation task: Participants are asked to detect whether the two lines (target) have the same orientation or not. **c** Digit categorization task: Participants must indicate whether the two digits (target) are concordant or discordant in their even–odd condition. In all cases, targets and distractors (emotional stimuli) appear (and disappear) at the same time

Experimental tasks such as those described in the Introduction (Fig. 1) consist of *concurrent but distinct target–distractor* (CDTD) paradigms (also named “directed attention tasks”; MacNamara, Kappenman, Black, Bress, & Hajcak, 2013). In other words, targets (i.e., elements on the screen to which endogenous attention must be directed to accomplish the task) and distractors (i.e., elements on the screen that are irrelevant to the task) are physically segregated and appear at the same time. Figure 3 illustrates some typical CDTD tasks. These paradigms are of great value for exploring exogenous attention, since they provide information on the three steps previously described: preattention, reorientation, and enhanced sensory processing.

Table 1 shows the main characteristics and conclusions of the 55 studies included in this review (see search methodologies in the next section). To the best of the author’s knowledge, there are no other studies that include the following characteristics: (1) A CDTD task is employed, and (2) neutral distractors are presented along with emotional distractors, −so that the emotion effect can be distinguished from that elicited by the mere presence of distractors. A version of this table is available online (www.uam.es/CEACO/sup/AtExogRev2013.htm), in which any potential study not present in the printed Table 1 but detected by readers will be added. Only data regarding exogenous attention are included in this review; several studies listed in Table 1 also explore endogenous attention to facial expressions or emotional scenes (which play the role of targets, instead of distractors, in some experimental conditions; see, e.g., Bishop, Duncan & Lawrence, 2004; MacNamara & Hajcak, 2010; Vuilleumier, Armony, Driver & Dolan, 2001), but this information is beyond the scope of this review.

**Table 1:** Description and main results of studies exploring exogenous attention to emotional stimuli employing CDTD tasks

Authors	Year	Sample Statistics: F/M (Average Age)	Sample Peculiarities	Ongoing Task	Average Accuracy in the Ongoing Task (0 to 100)	Nature of Distractors	Distractor Categories	Eccentricity of Distractors (Degrees from Fixation)	DVs Recorded	Any DV Signaled Emo > Neu?	Which Emo?	Any Modulating Factor?	First Emo > Neu Effects	Other Emo > Neu Effects	Brain Area Involved
Gilboa‐ Schechtman et al.	1999	Sample 1: 6/10 (31.6); Sample 2: 10/7 (34.12)	Sample 1: Social phobics. Sample 2: controls	Perceptual (visual search)	Not specified	Faces	4: Neutral, Negative, Positive	Peripheral, but not specified	Behavior	Yes, Behavior	Neg & Pos	Emotional content of targets			
Vuilleumier et al.	2001	6/6 (27.7)		Perceptual (faceshouses task)	84	Faces	2: Neutral, Negative	Inner edge when horizontal ≈ 1.6; Inner edge when vertical ≈ 0.625 (eccentricity not reported, but calculated from Figure 1)	Behavior, fMRI	Yes, Behavior & fMRI	Neg				(Mixed whole-brain & ROI strategy in the case of amygdala). Amygdala
Pessoa et al.	2002	8/13(22–38)		Perceptual (comparing orientation of bars)	64	Faces	3: Neutral, Negative, Positive	0	Behavior, fMRI	No					
Anderson et al.	2003	9/3 (22.1		Scene abstraction (Interior or exterior view of a house?)	87.3	Faces	3: Neutral, Fearful, Disgusted	0	Behavior, fMRI	Yes, fMRI	Neg (both Fearful and Disgusted)				(ROI strategy). Amygdala and anterior insula
Eimer et al.	2003	7/7 (29.6)		Perceptual (comparing line lengths)	≈ 97 (exact value not specified)	Faces	7: Neutral, Hapiness, Anger, Disgust, Fear, Sadness, Surprise	Center at 2.2	Behavior, ERPs	No					
Holmes et al.	2003	11/7 (23.7)		Perceptual (faceshouses task)	83.4	Faces	2: Neutral, Negative	Center at 2.5	Behavior, ERPs	No					
Fenske & Eastwood	2003	Exp. 2 (that relevant here): 48 participants, F/M proportion not specified (young adults, age not specified)		Perceptualemotional (recognizing the facial expression present in the target face)	96.36	Iconic symbols (≈facial emoticons)	3: Neutral, Negative, Positive	Peripheral, but not specified (distracter face ‐target/central face gap: 0.76)	Behavior	Yes, Behavior	Neg & Pos	Emotional content of targets			
Bishop et al.	2004	20/7(18–38)	Anxiety measured (trait and state )	Perceptual (faceshouses task)	Not specified	Faces	2: Neutral, Negative	Peripheral, but not specified	Behavior, fMRI	Yes, fMRI	Neg	Anxiety			(ROI strategy). Amygdala
Carretié et al.	2004	28/9 (21.54)		Perceptual (frame color changes)	95.97	Scenes	3: Neutral, Negative, Positive	0	ERPs	Yes, ERPs	Neg & Pos		≈100 ms (posterior P1)	anterior P2, N2	(Whole brain strategy). Occipital lobe, ACC
Harris & Pashler	2004	Exp. 2 (that relevant here): 124 participants, F/M proportion not specified (young adults, age not specified)		Digit categorization	Not specified	Words	2: Neutral, Negative	0	Behavior	Yes, Behavior	Neg	Distracter repetition			
Carretié et al.	2005	23/8(21.35)	Fear of spiders (used as negative stimuli)	Digit categorization	97.8	B/W silhouettes	2: Neutral, Negative	Inner edges at 17.2 horizontally, 12.35 vertically	Behavior, ERPs	Yes, ERPs	Neg		≈150 ms (anterior P150)	P500	(Whole brain strategy). vmPFC, precuneus, STG, PCC
Pessoa et al.	2005	7/13 (20–40)		Perceptual (comparing orientation of bars)	3 levels: Low difficulty (92), Medium (84), High (67)	Faces	2: Neutral, Negative	0	Behavior, fMRI	Yes, fMRI	Neg	Task difficulty			(Mixed whole brain & ROI strategy in the case of amygdala). Amygdala
Erthal et al.	2005	Exp. 1: 12/12 (21); Exp. 2: 18/18 (21.3); Exp. 3: 0/30 (22.3)	Sample 3: under the effects of alcohol	Perceptual (comparing orientation of bars)	Exp. 1: Low difficulty (93.6), Medium (86.7), High (78.7). Exp. 2: Low (94.9), Very High (61.1). Exp. 3: Low (94), Medium (90), High (82).	Scenes	2: Neutral, Negative	0	Behavior	Yes, Behavior	Neg	Task difficulty			
Schimmack	2005	Exp. 1: 63/63 (20); Exp. 2: 30/30 (young adults, age not specified).		Exp. 1: Arithmetical. Exp. 2: Perceptual (discriminating location of a line).	93	Scenes	7 unspecific: Neutral, Negative (3 arousal levels), Positive (3 arousal levels); 5 specific: Snakes, Faces (same or opposite sex), Bodies (same or opposite sex)	0	Behavior	Yes, Behavior	Neg & Pos	Target arousal			
Keil et al.	2005	7/4 (23.33)		Perceptual (detecting dot patterns)	90.7	Scenes	2: Neutral, Negative	Center at 3.9	Behavior, ERPs	Yes, Behavior & ERPs	Neg		Steady state paradigm: posterior SSVEP		
Holmes et al.	2006	8/4 (31)		Perceptual (comparing line lengths)	77.8	Faces	2: Neutral, Negative	0	Behavior, ERPs	Yes, ERPs	Neg		≈190 ms (anterior P2)		
Hahn et al.	2006	Exps. 2 and 3 (those relevant here): Sample 1 Exp 2: 6/8 (22.8); Sample 2 Exp 2: 7/7 (65.2); Sample 1 Exp 3: 8/7 (22.4); Sample 2 Exp 3: 8/7 (64.5)	Sample 1: young participants. Sample 2: old participants	Perceptualemotional (Exp. 2: detecting any discrepant face within an array of faces; Exp. 3: visual search of a specified facial expression within an array of faces)	From ≈ 89 to ≈ 100 (acc. provided only graphically)	Iconic symbols (≈facial emoticons)	3: Neutral, Negative, Positive	Peripheral, but not specified	Behavior	Yes, Behavior	Neg & Pos	Age			
Horstmann & Bauland	2006	Exp.1 (that relevant here): 6/6 (25)		Perceptualemotional (recognizing the facial expression present in the target face)	96	Iconic symbols (≈facial emoticons)	3: Neutral, Negative, Positive	Center at 1.2	Behavior	Yes, Behavior	Neg	Emotional content of targets			
Straube et al.	2006	Sample 1: 11/0 (20.9); Sample 2: 12/0 (21.3)	Sample 1: spider phobics. Sample 2: controls	Perceptual (line orientation discrimination)	≈ 94	Scenes	3: Neutral, negative (phobia‐related)	0	Behavior, fMRI	Yes, fMRI	Neg	Phobia			(ROI strategy). Amygdala
Bishop et al.	2007	10/8(27)	Anxiety measured (trait and state )	Perceptual (letter detection)	2 levels:Low difficulty: 93.65. High difficulty: 66.55	Faces	2: Neutral, Negative	0	Behavior, fMRI	Yes, Behavior & fMRI	Neg	Task difficulty and anxiety (only in fMRI, in the latter case)			(ROI strategy). Amygdala and STS for state anxiety, dlPFC (MFG) and ACC for trait anxiety
Aquino & Arnell	2007	6/7 (19.7)		Digit categorization	92.9	Words	4: Neutral, Threatrelated, Schoolrelated, Sexual	0	Behavior	Yes, Behavior	Sexual				
Silvert et al.	2007	7/3 (18–30)		Perceptual (a variant of the houseface paradigm in which orientation is also manipulated)	2 levels: Low difficulty: ≈94, High: ≈ 80 (acc. provided only graphically)	Faces	4: (Neutral, Negative) x (Easy, Difficult)	Center at 6.5	Behavior, fMRI	Yes, fMRI	Neg	Task difficulty (			(ROI strategy). Amygdala
Mitchell et al.	2007	9/6 (26.1)		Easy task: perceptual (case categorization); Difficult: lexical (syllable discrimination)	2 levels: Low difficulty (94.6), High (84.6)	Faces	2: Neutral, Negative	0	Behavior, fMRI	Yes, Behavior & fMRI	Neg	Task difficulty (only in fMRI)			(Mixed whole brain & ROI strategy in the case of amygdala). Superior occipital cortex, ventral lateral prefrontal cortex, ACC.
Hsu & Pessoa	2007	11/9 (19–29)		Perceptual (letter detection)	3 levels: Low difficulty (98), High "salience" (84.1), High "attentional load" (81.8).	Faces	2: Neutral, Negative	Center at 5	Behavior, fMRI	Yes, fMRI	Neg	Task difficulty (only in late trials)			(ROI strategy). Amygdala
Eimer & Kiss	2007	8/8 (29)		Perceptual (luminance changes in the fixation cross)	97.5	Faces	2: Neutral, Negative	Peripheral, but not specified	Behavior, ERPs	Yes, ERPs	Neg		≈200 ms (N2pc)		
Okon‐Singer et al.	2007	Exp. 1: 15/13 (25.07). Exp.2: 32/5 (22.86)		Perceptual (letter discrimination)	Exp. 1: 96.3; Exp. 2: 91.5	Scenes	2: Neutral, Negative	Exp. 1: Center at 7.5. Exp 2: 0.	Behavior	Yes, Behavior	Neg	Attentional resources availability			
Lim et al.	2008	12/17 (18–34)		Perceptual (letter detection)	2 leves: Low difficulty (90), High (72.9)	Faces	4: (Neutral, Negative) x (shock conditioned, unconditioned)	0	Behavior, fMRI	Yes, Behavior & fMRI	Neg & Shock conditioned	Task difficulty (only in fMRI)			(ROI strategy). Amygdala, ACC, fusiform gyrus, middle frontal gyrus; superior parietal lobule
Müller et al.	2008	5/5 (20–26)		Perceptual (detecting moving & flickering squares)	64.13 (during the first second)	Scenes	3: Neutral, Negative, Positive	0	Behavior, ERPs	Yes, Behavior & ERPs	Neg & Pos		steady state paradigm: posterior SSVEP		
Alpers et al.	2009	19/0 (22.5)	Spider phobia	Perceptual (animal identification)	91.06	B/W silhouettes	2: Neutral, negative (phobia‐related)	0	Behavior, fMRI	Yes, fMRI	Neg				(ROI strategy). mPFC, occipital lobe, hippocampus, insula, and thalamic structures.
Carretié et al.	2009	26/4 (23.89)	Fear of spiders and cockroaches (used as negative stimuli)	Digit categorization	87.94	B/W static and moving silhouettes	4: (Neutral, Negative) x (Static, Dynamic)	Inner edge ≈ 7 (moving) or ≈ 10.5 (static)	Behavior, ERPs	Yes, Behavior & ERPs	Neg Dynamic		≈100 ms (posterior P1)		
MacNamara & Hajcak	2009	33/16 (young adults, age not specified)	Anxiety measured (trait and state )	Perceptual (a variant of faceshouses task employing scenes instead of faces)	90.42	Scenes	2: Neutral, Negative	Peripheral, but not specified	Behavior, ERPs	Yes, Behavior	Neg				
De Cesarei et al.	2009	16/16 (25.33)		Perceptual (detecting a gap in a frame)	95	Scenes	9: (Neutral, Negative, Positive) x (0 eccentricity, 8.2 eccentricity, 16.4 eccentricity)	3 eccentricities: center at 0, 8.2, or 16.4	Behavior, ERPs	Yes, ERPs	Neg & Pos	Eccentricity	>400 ms (LPP)		
Nummenmaa et al.	2009	Exp. 3 (that relevant here): 10/5 (23)		Motor‐perceptual (sacadde to the new location of the fixation cross)	92 (fixation < 4º from target)	Scenes	3: Neutral, Negative, Positive	Inner edge ≈ 2.6	Behavior (ocular)	Yes, Behavior	Neg				
Buodo et al.	2010	Sample 1: 12/0 (22.5); Sample 2: 12/0 (23.23)	Sample 1: blood phobics. Sample 2: controls	Perceptual (luminance changes in the fixation cross)	96.94	Scenes	3: Neutral, Negative related to blood phobia, Negative unrelated.	Inner edges 5.4	Behavior, ERPs	Yes, Behavior & ERPs	Neg (both types)	Phobia and distracter relaton to phobia	≈200 ms (N2pc)		
Pourtois et al.	2010	0/1 (30)	Epileptic patient (electrodes implanted)	Perceptual (faceshouses task)	97	Faces	2: Neutral, Negative	Peripheral, but not specified	Behavior, Intracranial ERPs	Yes, Behavior & intracraneal ERPs	Neg		≈210 ms		(ROI strategy ‐ intracraneal recording‐). Amygdala
MacNamara & Hajcak	2010	Sample 1: 13/2 (33.53). Sample 2: 11/4 (31.73)	Sample 1: GAD. Sample 2: controls	Perceptual (a variant of faceshouses task employing scenes instead of faces)	84.8	Scenes	2: Neutral, Negative	Peripheral, but not specified	Behavior, ERPs	Yes, Behavior	Neg	GAD			
Calvo & Nummenmaa	2011	24/12 (19–23)		Perceptual (which side the happy face appeared?)	Ocular R: 81; Manual R: 94	Faces	6: Neutral, Sad, Angry, Fearful, Disgusted, Surprised	Inner edges 2.5	Behavior (ocular and manual)	Yes, Behavior (ocular and manual)	All (Disgust & surprise to the greatest extent)				
Hodsoll et al.	2011	Exps. 1–4 (those relevant here): 6/5 (27), 16/8 (26); 9/7 (26); 6/4 (26)		Perceptual (detecting target face inclination)	Exp. 1: 94; Exp. 2: 95.33; Exp. 3: 95.67; Exp. 4: 94 %	Faces	3: Neutral, Negative, Positive (taking the 5 experiments as a whole)	Center at 2.86	Behavior	Yes, Behavior	Neg & Pos				
Huang et al.	2011	Exp. 1: 11/12 (18–27). Exp. 3: 23 participants, F/M proportion not specified (18–25)		Perceptual (detecting the location of a dot within the target face)	Exp. 1: 99.15; Exp. 3: 94.5	Iconic symbols (≈facial emoticons)	3: Neutral, Negative, Positive	Center at 4.77	Behavior	Yes, Behavior	Neg	Attentional resources availability			
Carretié et al.	2011	21/5 (22.73)		Digit categorization	88.43	Scenes	3: Neutral, Fearful, Disgusting	0	Behavior, ERPs	Yes, Behavior & ERPs	Disgusting		≈200 ms (anterior P2)		(Whole brain strategy). Occipital lobe.
Wiens et al.	2011	7/7 (24)		Perceptual (letter detection)	88.5	Scenes	3: Neutral, Negative	0	Behavior, ERPs	Yes, ERPs	Neg		>400 ms (LPP)		
Barratt & Bundesen	2012	Exp. 1: 26/14 (21.1). Exp. 2: 15/15 (35.7)		Exp. 1: Perceptualemotional (recognizing the facial expression present in the target face). Exp. 2: Perceptual (discriminating letters).	Exp. 1: 93.32; Exp.2: 95.53	Iconic symbols (≈facial emoticons)	2: Neutral, Negative	Center of distracters at 7.8	Behavior	Yes, Behavior	Neg	Emotional content of targets and nature of the task			
Carretié et al.	2012	26/10(24)		Digit categorization	94.5	Scenes	9: (Neutral, Negative, Positive) x (High Spatial Frequency, Intact, Low Spatial Frequency)	0	Behavior, fMRI	Yes, Behavior & fMRI	Neg & Pos	Spatial frequency			(ROI strategy). Intraparietal sulcus (DAN), middle frontal gyrus (VAN & DAN)
Feng et al.	2012	13/13(21.69)		Perceptual (detecting color frame)	91.86	Scenes	4: Neutral, Negative, Positive (non erotic), Erotic	0	Behavior, ERPs	Yes, Behavior & ERPs	Erotic		≈200 ms (anterior P2)	N2, P3	
Lichtenstein‐ Vidne et al.	2012	50 participants in two experiments, F/M proportion not specified (young adults, age not specified)		Perceptual (indicating the location of the target, which was emotional in some conditions)	94 in both experiments	Scenes	3: Neutral, Negative, Positive	Peripheral, but not specified	Behavior	Yes, Behavior	Neg				
Nordström & Wiens	2012	16/15 (27)		Perceptual (letter detection)	≈94.5	Scenes	2: Neutral, Negative	0	Behavior, ERPs	Yes, ERPs	Neg		≈240 ms (LPN)	LPP	
Trauer et al.	2012	12/11 (23.4)		Perceptual (detecting moving & flickering squares)	92.3	Words	3: Neutral, Negative, Positive	0	Behavior, ERPs	Yes, ERPs	Neg		≈240 ms (anterior P2)		
Junhong, H. et al.	2013	Exp. 1: 24/11 (20.5). Exp. 2: 14/12 (20.8)		Lexical processing	Exp 1: Low difficulty (96.4), High (89.4). Exp 2: Low (96.6), High (94.5)	Faces	3: Neutral, Negative, Positive	Peripheral, but not specified	Behavior, ERPs	Yes, Behavior & ERPs	Neg & Pos (behavior), Neg (ERPs)		≈170 ms (anterior P2)		
López‐Martín et al.	2013	Sample 1: 0/20 (8–13); Sample 2: 0/20 (8–13)	Sample 1: ADHD, sample 2: controls	Digit categorization	Sample 1: 86; Sample 2: 90	Scenes	3: Neutral, Negative, Positive	0	Behavior, ERPs	Yes, Behavior & ERPs	Neg & Pos	ADHD	≈250 ms (N2ft)		
Syrjänen & Wiens	2013	17/17 (24.5)		Perceptual (letter detection)	Not specified	Scenes	3: Neutral, Negative, Positive	0	ERPs	Yes, ERPs	Neg & Pos	Gender	>400 ms (LPP)		
McSorley & van Reekum	2013	14/6 (19–21)		Motor‐perceptual (sacadde to the new location of the fixation cross)	81 (fixation < 2º from target)	Scenes	3: Neutral, Negative, Positive	Inner edges at 1	Behavior (ocular)	Yes, Behavior	Neg				
Schönwald & Müller	2013	13/7 (23.85)		Perceptual (detecting moving & flickering squares)	69.36	Scenes	2: Neutral, Negative	0	Behavior, ERPs	Yes, Behavior & ERPs	Neg		≈280 ms (EPN)	LPP	(Whole brain strategy). V1, lateral occipital gyrus, left occipito‐parietal areas, middle occipital, angular gyrus, lateral occipital temporal and superior temporal gyrus.
Carretié et al.	2013a	26/4 (19.65)		Digit categorization	93.29	B/W silhouettes	2: Neutral, Negative	3 eccentricities: inner border of the distracter at 0, 11.29, or 30.06	Behavior, ERPs	Yes, ERPs	Neg		≈240 ms (N2ft)		(ROI strategy). vPFC.
Carretié et al.	2013b	28/6 (22.79)		Digit categorization	88	Faces vs Scenes	6: (Neutral, Negative, Positive) x (Faces, Scenes)	0	Behavior, ERPs	Yes, Behavior & ERPs	Neg & Pos		≈180 ms (anterior P2 & N170)		(ROI strategy). Faces: Fusiform and IPL. Scenes: precentral gyrus.
Sussman et al.	2013	82/67(18.33)	Worry measured	Perceptual (dot color detection)	Not specified	Scenes	6: (Neutral, Negative, Positive) x (Low, High arousal)	Peripheral, but not specified	Behavior	Yes, Behavior	Neg	Worry			

*Note*. Studies in which authors are underlined are those providing information enough to be included in meta-analyses (see the main text). DV = dependent variable

In subsequent sections, we shall discuss the main data provided by these reports in detail, but a basic, conspicuous finding should be mentioned in advance: The vast majority of studies using CDTD tasks find some index (behavioral and/or neural) of exogenous attention bias toward emotional with respect to neutral distractors. This type of task is, therefore, an optimal and highly sensitive tool for exploring this process.

Studies in which emotional distractors and targets receiving endogenous attention are not physically segregated—such as those exploring the emotional Stroop effect (i.e., the categorization of the ink color in which the word is written is interfered with by its emotional content; e.g., Constantine, McNally & Hornig, 2001; Thomas, Johnstone & Gonsalvez, 2007), those using affective lexical decision tasks (word/pseudoword categorization is interfered with by the emotional content; e.g., Gutiérrez & Calvo, 2011; Kanske & Kotz, 2007; Kuchinke, Jacobs, Grubich, Vo et al., 2005), or those using tasks where specific nonemotional elements or categories (e.g., gender) within a face or scene have to be detected (detection is interfered with by the emotional content of the picture; e.g., Critchley, Daly, Phillips, Brammer et al., 2000; Eastwood, Smilek & Merikle, 2003; Morris, Friston, Büchel, Frith et al., 1998; Rellecke, Palazova, Sommer & Schacht, 2011; Simpson, Ongür, Akbudak, Conturo et al., 2000)—do not trigger evident spatial, VAN/DAN-related reorienting mechanisms, so that they will not be included. However, it is important to note that, globally, such studies yield results that are closely in line with those reviewed here, indicating greater interference of emotional content than of neutral content.

On the other hand, experimental paradigms in which targets and emotional distractors are not concurrent in time also provide key information on exogenous processes. Examples of these paradigms are those in which performance in the processing of targets in the ongoing, controlled task is modulated by a previously presented emotional cue in the same or a different location, as in the dot probe task (e.g., Brosch, Pourtois, Sander & Vuilleumier, 2011; MacLeod & Mathews, 1988), in affective variants of the cue–target Posner paradigm (Fox, Russo & Dutton, 2002; Pourtois, Grandjean, Sander & Vuilleumier, 2004), in the backward masking paradigm (Esteves & Öhman, 1993; Morris , Öhman, & Dolan, 1999; Ruiz-Padial & Vila, 2007), in affective attentional blink (Anderson & Phelps, 2001; Huang & Luo, 2007; Schwabe, Merz, Walter, Vaitl et al., 2011), in oddball paradigms that include emotional stimuli in the sequence of standard and deviant stimuli (Pannu Hayes, LaBar, Petty, McCarthy, & Morey, 2009), or in other experimental paradigms in which targets and distractors are also presented at different moments in time (Batty & Taylor, 2003; Pereira, Volchan, de Souza, de Oliveira et al., 2006; van Hooff, Crawford & Van Vugt, 2011; van Hooff, Devue, Vieweg & Theeuwes, 2013). However, the prior presentation of emotional stimuli with respect to target involves automatic engagement, a very important process that is beyond the scope of this review, due to limitations of space. In any case, and in line with the results presented here, these studies show that engagement with previously presented emotional visual stimuli can modulate attention to forthcoming ongoing-task-related targets even when the former are irrelevant to the task.

Finally, experimental paradigms in which the target and distractor are not defined a priori, since there is no explicit task directing endogenous attention to any particular element on the screen, also inform about the extent of the automaticity with which emotional stimuli access attentional resources. In these tasks, emotional stimuli are as relevant—or irrelevant—for the task as neutral ones. One example is binocular rivalry, in which different stimuli are simultaneously presented to each eye but only one of them usually reaches consciousness at a particular time. A number of studies have revealed that, during binocular rivalry, emotional stimuli preferentially access conscious perception with respect to neutral stimuli (e.g., Alpers & Pauli, 2006; Bannerman, Milders, De Gelder & Sahraie, 2008). This finding reinforces the idea of preferential automatic access to attentional resources by emotional stimuli. However, monocular dominance is observed even when two different *neutral* stimuli, such as grids with different orientations, are presented separately and simultaneously to each eye, so that binocular rivalry has been proposed to involve other processes besides exogenous attention (Blake, 2011); consequently, such studies will not be reviewed here.

#### Search and data description methodologies

Search of relevant studies was carried out through different tools and databases (PsychInfo©, Google Scholar©, PubMed©, ISI WoK©, and Scopus©, among other resources also involving book search) and employing diverse search terms. Due to the already mentioned nonstandardized nomenclature in this field of research, all papers including the words “attention” (with no specification of any attention type), “emotion,” and “target OR distracter OR distractor” were downloaded—or requested of authors—and read to detect whether, indeed, each of them described a relevant study. This search began in 2002, when the author designed his first experiment on exogenous attention to emotional stimuli (Carretié, Hinojosa, Martín-Loeches, Mercado, & Tapia, 2004), and has been systematic since 2011.

Data provided by selected studies (see the previous section) were submitted to meta-analysis when possible (i.e., 8 or more relevant studies relevant to a reviewed topic) and were qualitatively described in the rest of the cases. As a consequence, meta-analyses were carried out only on behavioral indices of exogenous attention (particularly, on reaction times, the most employed behavioral parameter in this field); as is described in the Neural Mechanisms section, neural indices were very diverse—involving different ERP components and different voxel coordinates—so the 8-study threshold was not reached in any of them. Cohen’s effect sizes (ESs), the parameter submitted to meta-analyses, consisted of standardized mean differences computed whenever one of the following numerical values regarding relevant contrasts was reported in the paper: Fischer’s *F* (obtained in one-way, two-level ANOVAs), means and dispersion measures, or Student’s *T* values. Calculation of ES from these three parameters required formulas for paired samples (e.g., Lakens, 2013), since all studies employed repeated measures designs to compare emotional versus neutral distractor effects. As is shown in Table 1, this information was available in 27 out of the 55 studies reviewed, which described 32 experiments (some studies described more than 1 experiment); the rest of the studies provided insufficient information to compute ES. Details and summaries on all present and absent values in each experiment are available at www.uam.es/CEACO/sup/AtExogRev2013.htm.

For global statistics on ES (i.e., calculation of mean ES and its statistical significance through a *Z* test; Lipsey & Wilson, 2001), the “MeanES” SPSS macro designed by Wilson (2010) was employed. To investigate potential moderators of ES, a *Q* statistic analog to analysis of variance (ANOVA) for categorical variables and a *Q* statistic modified weighted regression approach for continuous variables (Lipsey & Wilson, 2001) were computed also through Wilson’s SPSS macros (“metaF” and “metaREG,” respectively; links to these macros are available at http://www.uam.es/CEACO/sup/AtExogRev2013.htm). All analyses were conducted using maximum likelihood, random-effects models weighted by the inverse of the variance.

To address the “file drawer problem”—that is, the bias for significant results to be more likely published and retrievable for a meta-analysis, relative to nonsignificant results—the fail-safe *N* (*N*fs) was computed. This *N*fs represents the estimated number of unpublished studies reporting null results (here defined as ES = 0.1) that should exist to render the overall findings nonsignificant (Rosenthal, 1979). To this aim, the Orwin (1983) *N*fs formula was applied.

## Exogenous attention to emotional stimuli: main findings and modulating factors

### Main findings

As was already mentioned and as can be seen in Table 1, the majority of studies (92.73 %) found some indication (behavioral and/or neural) of greater exogenous attention to emotional than to neutral distractors. Behavioral indices (accuracy, reaction times, or ocular activity) were recorded in 53 studies (96.36 %), and neural signals (ERPs, fMRI, or intracranial recordings) were recorded in 38 studies (69.09 %), usually along with behavioral indices. In general, behavioral parameters were sensitive enough to detect significant differences between emotional and neutral distractors (66.04 % of the studies), but neural indices were the most sensitive (86.84 % of the studies among those analyzing brain activity). Indeed, in 17 studies (30.91 % of the total list), neural activity, but not behavioral activity (which was also recorded), detected exogenous attention biases toward emotional distractors.

**Fig. 4:**
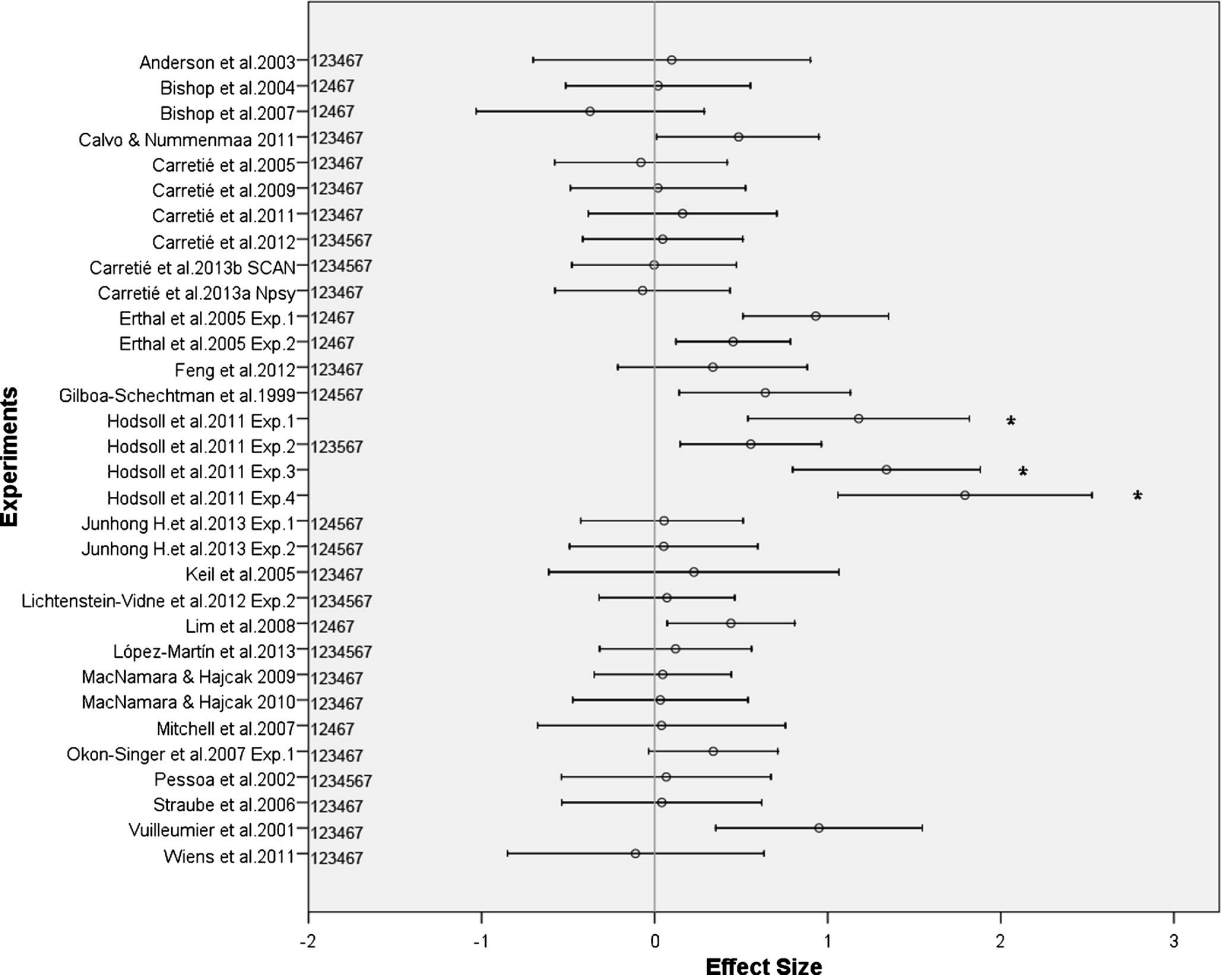
Experiments susceptible to being included in meta-analysis from those summarized in Table 1. Mean effect sizes (emotional minus neutral reaction times) and 95 % confidence intervals are shown. An outlier test recommended leaving studies marked with an asterisk out of the meta-analyses. Digits besides the experiments’ identification indicate the meta-analyses in which they were able to be included (details in different sections of the paper): 1 = Emo > Neu, 2 = Emo > Neu by Task, 3 = Emo > Neu by Accuracy; 4 = Neg > Neu; 5 = Pos > Neu, 6 = Emo > Neu by Distractor, 7 = Emo > Neu by eccentricity

However, a meta-analysis on reaction times in the ongoing CDTD task for studies in which numerical information was sufficient (see the Search and Data Description Methodologies section) confirmed that emotional versus neutral distractor ESs were significant also at the behavioral level. As was indicated in the Introduction, reaction times during the ongoing task increase as distractors capture more attentional resources in CDTD tasks. For example, longer reaction times to emotional than to neutral distractors mean that exogenous attention is greater to the former is than to the latter. Figure 4 shows ESs and 95 % confidence intervals (CIs) for meta-analyzed experiments (those with the label “1” in the figure; as may be appreciated, three experiments were detected to be outliers and were not included). Global computations showed that the mean ES for this sample of studies (mean ES = 0.223, 95 %CI = 0.113–0.333) was statistically significant (*Z* = 3.974, *p* < .001; *N*fs = 35.7), clearly supporting an emotional > neutral effect on exogenous attention.

Therefore, in quantitative terms, a conclusion can be drawn: Studies converge in indicating that emotional distractors capture attention to a greater extent than do neutral stimuli. However, this effect has been proposed to be modulated by certain factors that may reduce or even extinguish it. In this respect, it is important to stress at this point that endogenous, top-down attention is concerned to leave distractors out of the way of our limited conscious processing resources (Lavie, 2005). Only when distractors reach a certain saliency threshold might exogenous attention be devoted to them (Koster, Crombez, Van Damme, Verschuere, & De Houwer, 2004; Mogg & Bradley, 1998). It has been proposed that this threshold depends on several factors regarding the ongoing task (e.g., the level of involvement in the ongoing cognitive task; Pessoa & Ungerleider, 2004; Schwartz, Vuilleumier, Hutton, Maravita et al., 2005), the distractor (e.g., its valence or its facial/nonfacial nature; Carretié, Hinojosa, Martín-Loeches, Mercado et al., 2004; Carretié, Kessel, et al., 2013), and the individual’s state and trait characteristics (e.g., anxiety levels; Bishop, 2008; Mogg & Bradley, 1998). Current information on the modulatory effect of these factors is reviewed next.

### Modulatory effects of the ongoing task

#### Cognitive nature of the ongoing task

The nature of CDTD tasks is very variable from one study to another. As can be observed in Table 1, the majority of tasks employed up to now (80 %) involve only perceptual processing (comparing pictures, line lengths, line orientations, etc.). Another frequent task has been digit categorization (16.36 %). Finally, although less commonly employed (7.27 %), there are tasks requiring lexical decision, scene abstraction, or arithmetic processing (please note that some studies applied different tasks, so the sum is >100 %). Each of these tasks triggers distinct neural mechanisms, but when other factors are maintained constant, such as task difficulty (which is independent of the nature of task; it will be dealt with in the next section), global results on exogenous attention to emotional distractors appear to be similar. Thus, 90.91 % of perceptual tasks and 100 % of the rest of the tasks showed themselves to be more interfered with by emotional distractors than by neutral distractors at some level (behavioral and/or neural; at the behavioral level, exclusively, percentages were 61.36 % and 76.92 %, respectively).

However, in the particular case of behavioral indices of exogenous attention, a meta-analysis signaled significant differences among tasks. Thus, employing the *Q* statistic analog to ANOVA (see the Search and Data Description Methodologies section) on the emotional > neutral ESs regarding reaction times, the modulator role of type of task (two levels: perceptual vs. others) was analyzed. Twenty-nine studies were included in this analysis (those with the label “2” in Fig. 4). Results showed a significant effect of type of task, *Q*(1) = 7.099, *p* = .008. Mean ES for *perceptual* tasks (*n* = 19) was 0.317 (95 %CI = 0.209–0.425), greater than that for *other tasks* (*n* = 10), which was 0.050 (95 %CI = −0.115–0.214). This effect was not due to task difficulty: Perceptual and nonperceptual tasks did not differ in the accuracy reported, *F*(1, 21) = 0.71, *p* = .793. These unpredicted results suggest that, at the behavioral level, perceptual and nonperceptual CDTD tasks may be differentially affected by emotional distractors, the former being more susceptible to distractibility. The fact that target–distractor conflict is mainly produced in perceptual terms would probably be among the causes of this finding. In any case, nonperceptual studies are still scarce, so further research is needed to confirm these results and to reach firmer conclusions about this issue.

#### Attentional load in the ongoing task

Regardless of its cognitive nature, the task in which the individual is immersed while emotional distractors appear may compromise conscious, limited processing resources to very different extents. Under certain circumstances, the ongoing task exhausts these processing resources, so that irrelevant stimuli in the visual scene cannot be consciously perceived. This situation is known as attentional blindness (Mack & Rock, 1998; Simons, 2000; Simons & Ambinder, 2005), and its occurrence depends on the attentional load associated with the consciously processed event. Free resources for exogenous attention would be available only when the ongoing task is not sufficiently demanding (Lavie, 1995, 2005).

In CDTD tasks, information on the modulatory effects of task difficulty proceeds from two types of studies. First, several experiments have specifically manipulated the level of difficulty in the ongoing task, since difficulty and cognitive load positively correlate (Lavie, 1995, 2005). Two of them found greater *behavioral* indices of exogenous attention to negative distractors than to neutral distractors, but this effect was suppressed in the condition of maximal difficulty within each experiment (i.e., accuracy = 61.1 % in Erthal, De Oliveira, Mocaiber, Pereira et al., 2005, and 89.4 % in Junhong, Renlai & Senqi, 2013). However, two other studies manipulating difficulty level have not found significant modulations at the *behavioral* level negative distractors elicit greater indices of attentional capture than do neutral ones whatever the level of accuracy (Lim, Padmala & Pessoa, 2008; Mitchell, Nakic, Fridberg, Kamel et al., 2007).

Second, another set of experiments have not manipulated difficulty level but provide relevant information too. For example, experiments employing only very difficult tasks (with accuracies under 70 %) have also shown significantly greater *behavioral* indices of exogenous attention to emotional, as compared with neutral, distractors (Müller, Andersen & Keil, 2008; Schönwald & Müller, 2014). A meta-analysis was carried out with experiments belonging to this second set. To that aim, the weighted regression procedure described in the Search and Data Description Methodologies section was carried out using ESs regarding emotional > neutral reaction time ESs as the dependent variable and accuracy as the independent variable. Twenty-nine studies were able to be included in this analysis (those with the label “3” in Fig. 4). The association between ESs and accuracy was found to be far from significance, *R* ^2^ = .010, *β* = 0.099, *Z* = 0.416, *p* = .677.

Taken together, behavioral data suggest that emotional distractors may be capable of interfering with the ongoing task at very different levels of cognitive involvement. Some theoretical frameworks may provide an explanation for this conclusion, such as the processing efficiency theory (Eysenck & Calvo, 1992) or, more recently, the attentional control theory (Eysenck, Derakshan, Santos & Calvo, 2007). These theories propose that bottom-up attention to distractors increases as negative affect state (e.g., anxiety or stress) increases. These theories propose that, the prediction would be that highly demanding tasks, generally associated with enhanced subjective and physiological indices of negative affect state (e.g., Callister, Suwarno & Seals, 1992), would facilitate exogenous attention to distractors. In any case, and from an evolutionary point of view, the fact that emotional, biologically salient distractors capture attention also during highly demanding tasks seems a reasonable strategy.

A different panorama is observed when the focus of analysis is not behavior but activity at the neural level and, particularly, in the amygdala. There is an open debate on whether this structure is able to respond to emotional distractors in an automatic, mandatory fashion or whether its response depends on the availability of free processing resources. The first information on this issue proceeds from the study by Vuilleumier and colleagues (2001), which, employing the house–face task (see Fig. 3), described an enhanced amygdalar response to negative faces, as compared with neutral faces, also when they were distractors (attention to houses). The average difficulty of this task was intermediate (accuracy = 84 %). A year later, Pessoa, McKenna, Gutierrez and Ungerleider (2002) found no differential responses in the amygdala to neutral and negative distractors on employing a difficult task (accuracy = 64 %). Since then, several experiments manipulating the level of difficulty in the ongoing task have explored the amygdala’s responses to negative distractors. Results supporting the idea that these responses decrease with increased difficulty in CDTD tasks have been frequently reported (Bishop, Jenkins & Lawrence, 2007; Lim et al., 2008; Pessoa, Padmala & Morland, 2005; Silvert, Lepsien, Fragopanagos, Goolsby et al., 2007). These studies showed no amygdala bias toward emotional distractors with respect to neutral distractors when accuracies were under (or equal to) 80 %.

This behavioral versus neural (amygdalar) divergence regarding the effect of difficulty in CDTD tasks is probably due to the fact that behavior is the final single output of diverse neural discrete processes that may not always converge and that, case by case, may not always correlate with behavior. However, data exist suggesting that difficulty in the ongoing task may not be the single crucial factor explaining amygdala results. For example, similar amygdala responses to emotional and neutral distractors even in low-demanding tasks (accuracy > 90 %) have been reported (Alpers, 2009; Mitchell et al., 2007).

#### Emotion in the ongoing task

An interesting question is whether emotional distractors are capable of capturing attention even when targets also present affective charge. In real situations, emotional distractors (e.g., a predator) may appear when the individual is focusing endogenous attention on an affectively charged task (e.g., feeding). Four studies found enhanced behavioral indices of exogenous attention to emotional distractors when targets were also emotional (Gilboa-Schechtman, Foa & Amir, 1999; Lichtenstein-Vidne, Henik & Safadi, 2012; MacNamara & Hajcak, 2009, 2010). These results suggest that our nervous system is able to detect salient distractors even when the ongoing task is emotionally charged. However, using symbolic material (emoticons/simple drawings), several studies have provided different results up to now. Although, in these studies, emotional stimuli (both negative and positive) elicited behavioral indices of enhanced exogenous attention capture, this effect disappeared when targets consisted of negative symbols (Barratt & Bundesen, 2012; Fenske & Eastwood, 2003; Horstmann, Borgstedt & Heumann, 2006). Since, as is indicated later (see the Visual Category: Words, Faces, Scenes section), there are some potential limitations to using emoticons as emotional stimuli, the results in question should be considered with caution.

#### Conclusions and future directions

The number of studies relevant to each conclusion is shown in square brackets in all Conclusions and Future Directions sections.

1. The cognitive nature of the CDTD task (perceptual, digit categorization, etc.) may modulate behavioral indices of exogenous attention to emotional distractors [50; also supported by meta-analysis].
2. Difficulty of/involvement in the ongoing task has no marked effect on exogenous attention to emotional distractors at the behavioral level and, clearly, is not a key factor explaining nonsignificant emotional versus neutral differences [49; also supported by meta-analysis].
3. Difficulty of/involvement in the ongoing task seems to cause stronger effects at the neural level, and particularly with respect to the amygdala, its activity in response to negative distractors decreases as difficulty in the ongoing task increases [14].
4. Emotional distractors capture attention even when the ongoing task is affectively charged if emotional pictures are employed as targets, but results could be different when symbols are used [8].

##### Future directions

Point 1 summarizes an unpredicted finding that requires further research, since nonperceptual tasks have been scarcely explored. Point 4 has also received scant attention, and, in general, neural mechanisms (besides the amygdala), in all points, are worth systematically exploring.

### Modulatory effects of individual state-trait characteristics

#### Anxiety

Several cognitive theories (Eysenck, 1992; Mathews, 1990; Mogg & Bradley, 1998; Williams, Watts, MacLeod & Mathews, 1997) defend an enhanced attentional bias toward negative stimuli in anxious individuals. Although these theories do not propose specific or explicit hypotheses on exogenous attention, several studies explore any potential bias affecting it in anxious individuals. Importantly, biases toward negative distractors could be potentiated by the impaired functioning of endogenous attention to the ongoing task that, according to the attention control theory (already mentioned in the Attentional Load in the Ongoing Task section), characterizes anxiety (Eysenck et al., 2007).

Part of the CDTD experiments in this field have focused on individuals experiencing subclinical unspecific anxiety (state and/or trait anxiety; Bishop et al., 2004; Bishop et al., 2007; MacNamara & Hajcak, 2009). At the behavioral level, these studies reported lack of significant differences with respect to low-anxious participants. In contrast, clinical unspecific anxiety (generalized anxiety disorder, or GAD) has been reported to be associated with significantly greater behavioral indices of exogenous attention to negative distractors (higher indices in GAD patients than in healthy controls; MacNamara & Hajcak, 2010). Therefore, and as is suggested by the scarce data currently available, unspecific anxiety needs to reach the clinical level in order to result in enhanced *behavioral* indices of exogenous attention to negative stimuli, at least when CDTD tasks are employed. However, *neural* activity appears to be more sensitive than behavior: Greater activity in the amygdala is observed in response to negative distractors in subclinical anxious, as compared with nonanxious, participants (Bishop et al., 2004; Bishop et al., 2007). Spatial localization of neural activity in clinical generalized anxiety has not yet been explored through CDTD tasks, but other studies exploring automatic processes triggered by masked (unconscious) stimuli have shown enhanced amygdalar reactions to negative stimuli in GAD patients, as compared with controls (Monk, 2008).

Specific anxiety (i.e., phobias) has also been explored with respect to exogenous attention. Three studies focusing on blood phobia (Buodo, Sarlo & Munafò, 2010), social phobia (Gilboa-Schechtman et al., 1999), and spider phobia (Straube, Mentzel & Miltner, 2006) have been carried out, all using CDTD tasks. Behavioral indices of enhanced attentional capture in phobic, as compared with nonphobic, participants by distractors related to their fear have been observed in the former two studies, but no differences were reported in the latter. At the neural level, and in temporal terms, differential responses between phobics and control participants are evident as early as approximately 200 ms (Buodo et al., 2010). In spatial terms, the phobic versus neutral distractor differential response was greater in the phobic sample amygdala than in the control sample amygdala (Straube et al., 2006).

#### Other individual characteristics

Some incipient data suggest that other traits and diseases besides anxiety may also modulate the threshold above which distractors capture attention. One of these is attention deficit hyperactivity disorder (ADHD), a condition in which one of the key symptoms is distractibility (American Psychiatric Association, 2013). A recent CDTD study explored whether this distractibility is biased toward emotional stimulation (López-Martín, Albert, Fernández-Jaén & Carretié, 2013). The data showed both behavioral and electrophysiological indices of enhanced exogenous attention to emotional distractors (both positive and negative) in ADHD boys, as compared with healthy controls. The opposite effect is hypothesized to occur in psychopathic individuals, who are suggested to be less prone to distractibility than are nonpsychopaths (Hiatt & Newman, 2006). Consequently, it has been posited that they should manifest reduced exogenous attention to emotional distractors (Blair, 2009). Experimental data on this issue are not available yet, but this theoretical proposal makes psychopaths a relevant target for future studies employing CDTD tasks. Finally, certain demographic factors probably modulate the capture threshold, although experimental data are almost nonexistent in this area. For example, gender (Syrjänen & Wiens, 2013) and age (Hahn, Carlson, Singer & Gronlund, 2006) have also been reported to modulate exogenous attention to emotional distractors.

#### Conclusions and future directions

1. In unspecific anxiety, behavioral indices of exogenous attention to negative distractors show greater effects when symptoms reach the “clinical” threshold. In specific anxiety (phobias), there is mixed behavioral evidence regarding exogenous attention to negative distractors [9].
2. Amygdala activity in response to negative distractors is enhanced by both unspecific and specific anxiety [3].
3. Other individual trait characteristics, such as ADHD or psychopathy, and demographic factors, such as gender or age, scarcely studied to date, may also modulate exogenous attention to emotional stimuli [3].

##### Future directions

In general, this promising area of research is understudied. In this respect, going beyond traditional clinical categorizations seems advisable. Indeed, different types of clinical and nonclinical anxiety may share (among themselves and also with other affective diseases) considerable variance on certain underlying dimensional constructs. For example, “fear and distress disorders” have been proposed to underlie several traditional anxiety and depression categories (Clark & Watson, 2006), and, interestingly, variation along such dimensions appears to modulate attention to emotional stimuli (Waters, Bradley & Mogg, 2014). These and other underlying dimensions are worth further exploring with respect to exogenous attention.

### Modulatory effects of the nature of emotional distractors

#### Affective content: valence, arousal, and beyond

Valence (ranging from negative or unpleasant to positive or pleasant) and arousal (ranging from calming to arousing) are two theoretically orthogonal affective dimensions widely considered to explain the principal variance of emotional meaning (Lang, Greenwald, Bradley & Hamm, 1993; Osgood, Suci & Tannenbaum, 1957; Russell, 1979; C. A. Smith & Ellsworth, 1985). With respect to valence, it has been proposed that negative events require processing and response resources to be intensely and urgently mobilized. Such urgency would have obvious adaptive and evolutionary advantages: The consequences of a negative event are often dramatic (Ekman, 1992; Öhman, Hamm & Hugdahl, 2000). Indeed, several studies indicate that negative events elicit more rapid and more prominent responses than do neutral or even positive events. This “negativity bias” is manifested at several cognitive levels, including the attentional level, and has been supported by several top-down attention studies (see a review in Carretié, Albert, López-Martín, & Tapia, 2009).

As can be appreciated in Table 1, 90.91 % studies show negative > neutral differences also in exogenous attention experiments. However, data on the negativity bias are conditioned by a sort of “experimental negativity bias”: Whereas *all* studies present negative distractors, only 23 studies (41.82 %) present positive distractors too. Among the latter studies, which are especially relevant here since they allow valence effects to be distinguished from arousal effects, negative distractors, and not positive ones, elicited higher indices of attentional capture than did neutral distractors in 6 studies (Horstmann et al., 2006; Huang, Chang & Chen, 2011; Lichtenstein-Vidne et al., 2012; McSorley & van Reekum, 2013; Nummenmaa, Hyona & Calvo, 2009; Sussman, Heller, Miller & Mohanty, 2013) and, along with positive distractors, in 13 (Carretié et al., 2004; Carretié, Kessel, et al., 2013; Carretié, Rios, Periáñez, Kessel, & Álvarez-Linera, 2012; De Cesarei, Codispoti & Schupp, 2009; Fenske & Eastwood, 2003; Gilboa-Schechtman et al., 1999; Hahn et al., 2006; Hodsoll, Viding & Lavie, 2011; Junhong et al., 2013; López-Martin et al., 2013; Müller et al., 2008; Schimmack & Derryberry, 2005; Syrjänen & Wiens, 2013). Two studies showed greater exogenous attention to positive stimuli, and not to negative stimuli, than to neutral stimuli (Aquino & Arnell, 2007; Feng, Wang, Wang, Gu, & Luo, 2012), and in both cases, positive stimuli were of sexual content (the remaining two experiments—Eimer, Holmes & McGlone, 2003; Pessoa et al., 2002—are among those not showing any differential effect of emotional distractors with respect to neutral).

To further explore this issue, meta-analyses on reaction times in the ongoing task were carried out separately for negative > neutral ESs (*n* = 28; those with the label “4” in Fig. 4) and for positive > neutral ESs (*n* = 10; label “5” in Fig. 4). Statistical tests showed that the mean effect size was greater in the negative > neutral analysis (mean ES = 0.229, 95 %CI = 0.116–0.342) than in the positive > neutral analysis (mean ES = 0.193, 95 %CI = 0.044–0.342), but, importantly, both were significant (*Z* = 3.971, *p* < .001, and *Z* = 2.532, *p* = .011, respectively). Thus, data available up to the moment show the enhanced capability of both negative and positive distractors to capture attention, but a certain advantage of the former cannot be discarded.

In experiments employing both negative and positive distractors, along with neutral, whether the superiority of negative stimuli in capturing attention—when it is observed—is exclusively due to their valence is, however, debatable. Negative stimuli often present greater arousal values than do positive. Thus, even when they are selected as equivalent in normative arousal ratings, experimental samples may assess the former as more arousing (see, e.g., Weinberg & Hajcak, 2010). An advisable strategy would be to analyze the actual statistical association of valence and arousal assessments of the stimuli—provided by the experimental sample itself—with the observed results (e.g., through multiple regression techniques). Indeed, a modulating role of arousal has been observed in exogenous attention studies employing CDTD tasks, although this dimension has been much less widely studied. In the two studies in which this factor was manipulated (Schimmack & Derryberry, 2005; Sussman et al., 2013), high-arousing emotional distractors elicited stronger behavioral indices of exogenous attention capture than did low-arousing distractors.

Some data suggest that studying exogenous attention beyond the valence × arousal (*circumplex*) frame can provide highly relevant information. Thus, within both “negative stimuli” and “positive stimuli,” subclassifications can be made. For example, several experiments have shown behavioral differences between the automatic processing of fearful and disgusting stimuli (Charash & McKay, 2002; Cisler, Olatunji, Lohr & Williams, 2009; van Hooff et al., 2013), despite the fact that these two emotions share the same emotional valence (negative) and have high ability to arouse (higher than that elicited by other negative emotions, such as sadness; Russell, 1980). This issue was recently explored through a CDTD task (Carretié, Ruiz-Padial, López-Martín & Albert, 2011), and an advantage was found for disgusting events, in line with Charash and McKay and van Hooff et al. (2013) (see Table 1). At the individual level, and as explained above (see the Anxiety section), certain specific types of negative distractors, such as those related to particular fears or phobias, elicit enhanced indices of exogenous attention, as compared with other unpleasant stimuli (Buodo et al., 2010; Straube et al., 2006). On the positive side of the valence dimension, there are also data showing enhanced capacity to capture attention by sexually-loaded distractors with respect to other positive stimuli (Feng, Wang, Wang, Gu et al., 2012). All these data suggest that the dimensional approach in the study of emotion, which relies on the idea that emotional states are well explained by valence and arousal, and the discrete approach, which defends the study of each emotion separately (e.g., Ekman, 1992; Izard, 1992; Panksepp, 1982), are both necessary and complementary for accounting for the effect of negative emotion on automatic attention.

#### Visual category: words, faces, scenes

The rich variety of emotional stimuli that humans process in their everyday life has been classified by experimental practice according to their categorial nature. Within the visual modality, stimuli can be divided into symbolic (e.g., written emotional words, signs, or simple drawings) and nonsymbolic material. The latter can be further subdivided into facial and nonfacial emotional scenes.

*Symbolic* material has been much less explored than nonsymbolic material as regards exogenous attention to emotional distractors. Only three studies employed words as distractors in CDTD tasks (Table 1), so only tentative conclusions can be extracted about their capacity for capturing attention. These studies suggest that emotional words capture attention to a lesser extent than does pictorial material. Thus, in the study by Harris and Pashler (2004), behavioral indications of exogenous attention to negative and neutral words were found only after their first presentation, and not in subsequent ones. Trauer, Andersen, Kotz and Müller (2012) reported ERP differences between negative and neutral distractors, although they attributed them to lexico-semantic processes, rather than to attention. Finally, Aquino and Arnell (2007) reported differences between sexually related items and neutral items, but not between threat-related or school-related items and neutral words.

These results are in line with those observed in other tasks, such as emotional Stroop or affective lexical decision: Interference of emotional words with respect to neutral words occurs only when they are especially intense (e.g., taboo words or insults; Baas, 2004; Carretié, Hinojosa, Albert, López-Martín et al., 2008; MacKay, Shafto, Taylor, Marian et al., 2004; Pratto & John, 1991) or when participants present affective disorders such as anxiety, depression, or posttraumatic stress disorder (for emotional Stroop, see reviews by Cisler, Wolitzky-Taylor, Adams, Babson et al., 2011; Whalen, Bush, Shin & Rauch, 2006; Williams, Mathews & MacLeod, 1996; for affective lexical decision, see Kanske & Kotz, 2007; Kuchinke et al., 2005; Nakic, Smith, Busis, Vythilingam, & Blair, 2006; Siegle, Ingram & Matt, 2002). The relatively weak capacity of emotional words to capture attention when they play the role of distractors is probably related to the suggestion (from research) that verbal emotional material is less arousing than other types of visual affective items, such as facial expressions or emotional scenes (Frühholz, Jellinghaus & Herrmann, 2011; Hinojosa, Carretié, Valcárcel, Méndez-Bértolo, & Pozo, 2009; Keil, 2006; Kissler, Assadollahi & Herbert, 2006; Mogg & Bradley, 1998; Okon-Singer, Lichtenstein-Vidne & Cohen, 2013; Vanderploeg, Brown & Marsh, 1987). Up to the present, CDTD tasks have not been employed for directly exploring this symbolic versus nonsymbolic distinction.

A remark should be made on studies employing iconic symbols. In all cases, they have consisted of simple facial line drawings (i.e., emoticons; Barratt & Bundesen, 2012; Fenske & Eastwood, 2003; Hahn et al., 2006; Horstmann et al., 2006). They all report exogenous attention biases toward emotional symbols, an “angry face advantage” being reported. However, Horstmann (e.g., Horstmann et al., 2006) has demonstrated a substantial influence of perceptual differences between the stimuli (rather than, or together with, emotional differences) in the observed results. Therefore, simple drawing results should be cautiously considered, and perceptual influences should be more systematically explored in the future.

In the case of *nonsymbolic* stimuli, and as specified in Table 1, both facial and nonfacial distractors have consistently been found to interfere with the ongoing task. A meta-analysis employing the *Q* statistic analog to ANOVA (see the Search and Data Description Methodologies section) was carried out on the emotional > neutral ESs regarding reaction times contrasting the modulator role of visual category of distractor (two levels: face vs. scene). Twenty-eight studies were able to be included in this analysis (those with the label “6” in Fig. 4). Results showed nonsignificant differences, Q(1) = 1.312, *p* = .252. Mean ES for faces (*n* = 12) was 0.314 (95 %CI = 0.165–0.464), and for scenes (*n* = 16), it was 0.203 (95 %CI = −0.087–0.320). These results suggest that, at least at the behavioral level, emotional facial and nonfacial stimuli capture attention to a similar extent. At the neural level, and as indicated in the Visual Category: Words, Faces, Scenes section, both types of emotional distractors are also associated with enhanced indices of exogenous attention. However, faces elicit temporally and spatially specific neural responses (e.g., involve the fusiform face area and elicit a specific ERP component, N170), so important qualitative differences between the cerebral response to facial and nonfacial distractors in CDTD tasks usually emerge (see Carretié, Kessel, et al., 2013, directly comparing both types of distractors).

#### “Magnocellular–parvocellular balance”: motion, spatial frequency, eccentricity

The visual route from the retina to the striate cortex consists of two parallel streams, the magnocellular and the parvocellular pathways. They originate from different retinal ganglion cells (Perry, Oehler & Cowey, 1984), which project to separate layers of the lateral geniculate nucleus (LGN) of the thalamus (Livingstone & Hubel, 1987). Magnocellular and parvocellular LGN neurons also project to separate layers of the striate cortex (Hubel & Wiesel, 1972). Then, parvo- and magnocellular inputs are integrated in the extrastriate cortex, although they are preferentially—not exclusively—associated with the ventral and dorsal cortical streams, respectively (Felleman & Van Essen, 1991; Merigan & Maunsell, 1993). Functionally, parvocellular and magnocellular systems differ in several aspects. The former is sensitive to color, higher spatial frequencies, and lower temporal frequencies and has lower contrast sensitivity; the magnocellular system is insensitive to color, responds to lower spatial frequencies and to higher temporal frequencies, and has higher contrast sensitivity (Derrington & Lennie, 1984; Schiller & Malpeli, 1978). Moreover, differences exist regarding the spatial location of the visual input characterized by an overrepresentation of central vision in the parvocellular pathway: Parvocellular to magnocellular ratios decrease from 35:1 at the fovea to 5:1 at 15° eccentricity (Azzopardi, Jones & Cowey, 1999). Finally, motion is also a characteristic differentially represented in both visual pathways: The magnocellular pathway provides the major input to cortical areas responsible for motion processing (DeYoe & Van Essen, 1988; Maunsell & Newsome, 1987).

A sort of “magnocellular–parvocellular balance” might, therefore, be established for distractors. Research on CDTD tasks employing only nonemotional stimuli show that parvocellular-biased distractors, such as isoluminant color changes, are not capable of capturing attention (Irwin, Colcombe, Kramer & Hahn, 2000; Theeuwes, 1995). Although this area of research has not been systematically studied to date in experiments including emotional distractors, some parameters are being incipiently explored, such as spatial frequency, motion, and eccentricity. Thus, centrally presented, unfiltered (i.e., all spatial frequencies present), and static distractors would be more parvocellular balanced, while, at the other extreme, peripherally presented, low-pass filtered (i.e., high spatial frequencies—details—are eliminated), and dynamic distractors would be more magnocellular balanced.

As regards *eccentricity* (measured by visual angle with respect to the fixation point), the initial, basic question that arises is whether our nervous system is actually capable of evaluating the emotional content of peripheral stimuli and, consequently, of triggering enhanced exogenous attention to peripheral emotional distractors. As can be seen in Table 1, 54.55 % of the studies have presented eccentric distractors (i.e., deviated from the central, foveally projected, area of the screen). A meta-analysis employing the *Q* statistic analog to ANOVA (see the Search and Data Description Methodologies section) was carried out on ESs regarding emotional minus neutral reaction times and contrasting the modulator role of distractor eccentricity (two levels: central vs. peripheral). Twenty-eight studies were able to be included in this analysis (those with the label “7” in Fig. 4). Results showed nonsignificant differences, *Q*(1) < 0.060, *p* = .807. Mean ES for central distractors (*n* = 15) was 0.235 (95 %CI = 0.107–0.364), and for peripheral (*n* = 13), it was 0.258 (95 %CI = 0.127–0.389). Therefore, emotional distractors do not lose their capability to capture attention when peripherally presented.

With respect to *spatial frequency*, enhanced top-down attention has been shown even when high spatial frequencies and color information have been eliminated (Alorda, Serrano-Pedraza, Campos-Bueno, Sierra-Vázquez, & Montoya, 2007; Carretié, Hinojosa, López-Martín & Tapia, 2007; Vuilleumier, Armony, Driver & Dolan, 2003). Data on this issue regarding exogenous attention are still very scarce, but one CDTD study providing structural connectivity and hemodynamic data support the key role of low spatial frequencies in attentional capture by salient/emotional distractors (Carretié, Ríos, Periáñez, Kessel et al., 2012).

Finally, as regards to *motion*, and despite the fact that emotional events are often dynamic in real-life situations, data on exogenous (but also endogenous) attention to moving emotional stimuli are surprisingly scarce. Dynamic nonemotional stimuli are detected more easily and more quickly than static stimuli, and an advantage of motion over other physical features, such as luminance or color, for capturing attention has been demonstrated (Franconeri & Simons, 2003, 2005). Interestingly, as can be seen in Table 1, this effect is enhanced when distractors present emotional charge along with motion: Dynamic negative distractors capture attention to a greater extent than do dynamic nonemotional events and than do static emotional events (Carretié, Hinojosa, Carretié, Valcárcel, Méndez-Bértolo et al., 2009).

#### Conclusions and future directions

1. Both negative and positive stimuli show greater exogenous attention capture than do nonemotional stimuli, a mild superiority of negative stimuli being observed in this respect [23; also supported by meta-analyses].
2. Specific content within “positivity” (e.g., sexual) and “negativity” (e.g., disgusting) causes specific attentional patterns that may not be attributable to valence and arousal [4].
3. Facial and nonfacial emotional distractors are similar as regards capacity for capturing exogenous attention [44; also supported by meta-analysis].
4. Studies on magnocellular- versus parvocellular-biased distractor characteristics suggest that exogenous attention may significantly rely on the magnocellular visual pathway [30; also supported by meta-analysis].

##### Future directions

Point 1 needs further exploration with respect to the arousal dimension, and point 2 would benefit from future studies exploring relevant specific emotional contents. With respect to point 3, direct studies comparing the capability of symbolic versus nonsymbolic emotional distractors to capture attention are necessary. Finally, as regards point 4, magnocellular-biased attributes, such as temporal briefness of distractors—usually associated with unconscious perception—motion, or others, scarcely explored up to date, would contribute to advance in the characterization of exogenous attention.

## Neural mechanisms

Once the superiority of emotional stimuli in capturing attention has been exposed, as well as the role of factors proposed to modulate this emotional advantage, the next step is to describe the mechanisms explaining the observed results. In this respect, neural information is crucial. Part of the experiments employing CDTD tasks (69.09 %) have recorded brain activity along with behavioral responses. Neural data both at the temporal and at the spatial level are necessary for a general understanding of the exogenous attention architecture.

### Temporal characterization: main phases

**Fig. 5:**
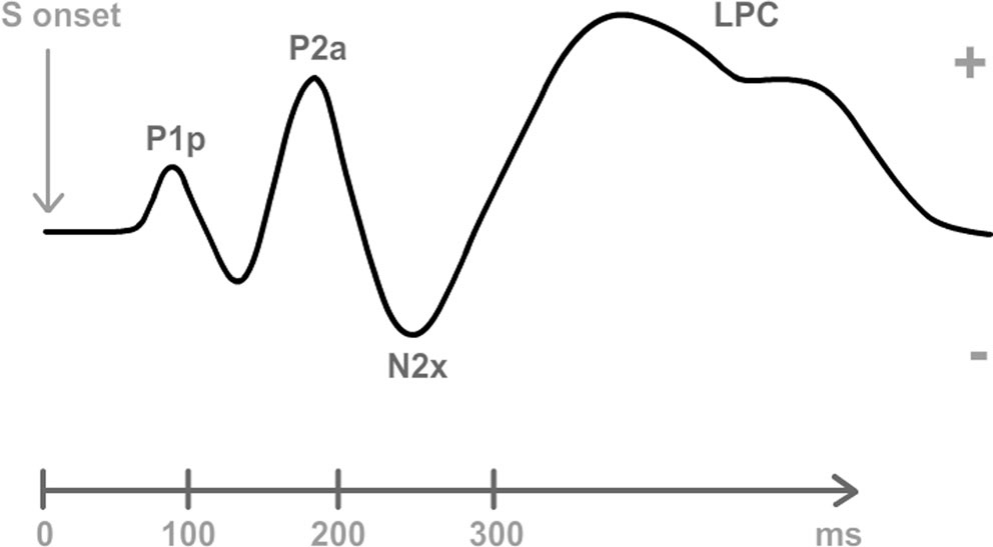
Graphical schematic summary showing the time-course of ERP components reported up to date to reflect exogenous attention to emotional distractors in concurrent but distinct target–distractor tasks

Information on neural timing is provided by 24 ERP experiments (Table 1). It is important to note that the majority of them followed a window-of-interest strategy and, therefore, did not analyze all ERP components (so that there may have been some effects that went unreported). Although data are still scarce and the temporal characterization of exogenous attention needs additional research, the effects reported to date allow us to draw a tentative picture of the temporal dynamics of exogenous attention to affective distractors, which is summarized in Fig. 5. At first glance, an interesting temporal characteristic emerges: exogenous attention to emotional stimuli triggers rapid neural responses. Thus, 41.67 % of ERP studies report enhanced amplitudes to emotional, as compared with neutral, distractors within the first 200 ms from target and distractor onset. The first effects have been observed at posterior P1 (P1p, peaking at 100 ms approximately, but with earlier onset), which has shown emotional > neutral amplitudes in two studies so far (Carretié Hinojosa et al., 2009; Carretié et al., 2005). In studies employing only nonemotional stimuli, P1 has indeed been proposed as reflecting exogenous attention (Hopfinger & Mangun, 2001). Early emotional effects (C1 component, peaking around 90–100 ms) have also been observed in tasks other than CDTD (see a review in Pourtois et al., 2012). In both cases, the evidence supports the idea of the nervous system’s capacity to rapidly evaluate the biological saliency of stimulation.

These components (P1 and C1) originate in the occipital cortex (Di Russo et al., 2002), so that they are probably reflecting sensory amplification. This exogenous subprocess is, therefore, not the final step of a serial sequence but may occur, at least partially, in parallel with other subprocesses (i.e., preattention/evaluation and reorienting). Initial preattention/evaluation processes are difficult to record through ERPs, not only because they compete in the same time window with intense perceptual processes (reflected in, relatively, very strong electrophysiological responses), but also because part of them probably originate in areas to which EEG is not sensitive enough or simply blind (as in the case of the amygdala, an electrically closed-field structure). A detailed discussion on these structures and their latencies using deep recordings (not affected by electrical field competition) is provided in the Preattention/Evaluation Network section.

An interesting finding revealed by the studies reviewed here is that the components showing maximal sensitivity to exogenous attention (in terms of number of ERP studies reporting it) are anterior P2 (P2a, peak at about 180–200 ms; Carretié et al., 2004, 2005; Carretié, Kessel, et al., 2013; Carretié et al., 2011; Feng et al., 2012; Holmes, Kiss & Eimer, 2006; Junhong et al., 2013) and the family of N2 components (N2, N2pc, N2ft (frontotemporal), peaking at 200–250 ms, approximately; Buodo et al., 2010; Carretié, Albert, López-Martin, et al., 2013; Carretié et al., 2004; Eimer & Kiss, 2007; Feng et al., 2012; López-Martin et al., 2013). The N2 family (N2x) should be distinguished from EPN (early posterior negativity), an emotion-sensitive component that always presents posterior distribution (this is not the case for N2x, as explained below) and relatively longer latencies and which has been reported to disappear when emotional stimuli act as distractors instead of as targets (e.g., Wiens, Sand, Norberg & Andersson, 2011; but see Schönwald & Müller, 2014). Source localization analyses on P2a and N2x indicate distinct origins, some of them located in cortical areas posited to intervene in preattention/evaluation (Carretié, Albert, et al., 2013; Carretié et al., 2005), in visual cortices probably reflecting sensory amplification (Carretié et al., 2004; Carretié, Kessel, et al., 2013; Schönwald & Müller, 2014), and in other structures belonging to VAN/DAN circuits involved in the reorienting of attention (Carretié, Albert, López-Martin, et al., 2013; Carretié et al., 2005; Carretié, Kessel, et al., 2013; Schönwald & Müller, 2014).

Finally, significant emotional > neutral effects have also been observed in CDTD tasks at late latencies in relation to different positivities occurring after 300 ms, here referred to as the late positive complex, or LPC (Carretié et al., 2005; De Cesarei et al., 2009; Feng et al., 2012; Nordström & Wiens, 2012; Schönwald & Müller, 2014; Syrjänen & Wiens, 2013; Wiens et al., 2011). These components have been proposed as significantly influenced also by top-down processes. For example, late positive potential (LPP)—the most important within the LPC in terms of number of studies reporting significant emotional > neutral effects—shows reduced amplitude in response to negative stimuli after their reinterpretation as less negative (Hajcak & Nieuwenhuis, 2006), when the attention within a negative picture is voluntarily directed toward less negative locations of the scene (Dunning & Hajcak, 2009) or when negative stimuli appear at endogenously unattended spatial locations (MacNamara & Hajcak, 2009). Whereas the influence of automatic processes on LPC should not be discarded (Hajcak , Dunning, & Foti, 2009), their latency and origin in CDTD tasks, mainly visual (Carretié et al., 2005; Schönwald & Müller, 2014), leads to the hypothesis that this complex is significantly reflecting endogenous, conscious—mainly spatial—attention to the distractor (see a review in MacNamara, Kappenman, Black, Bress et al., 2013).

Importantly, whether each of these components presents significant effects (or even whether they are present or not in the ERP) from one study to another appears to depend on several factors, critical among which is the spatial location of stimuli. In nonemotional studies, P2a has been shown to appear when targets are close to fixation, but not when they appear on the periphery (O'Connell, 2011). The N2 family (N2x, in Fig. 5) is also clearly influenced by spatial location. For example, N2pc appears only in response to lateralized stimuli, being maximal at the contralateral parietal scalp (Eimer, 1996; Luck, 1994), and N2ft, being maximal at frontal and temporal regions, is maximal when distractors present 0° eccentricity (Carretié, Albert, et al., 2013). P1 also depends on the physical location of the stimulation, its amplitude being higher in response to stimuli presented in the lower part of the visual field (Fortune & Hood, 2003). Therefore, the physical distribution of stimulation is a key factor to be taken into account when designing ERP studies exploring exogenous attention.

### Spatial characterization: main brain areas

**Fig. 6:**
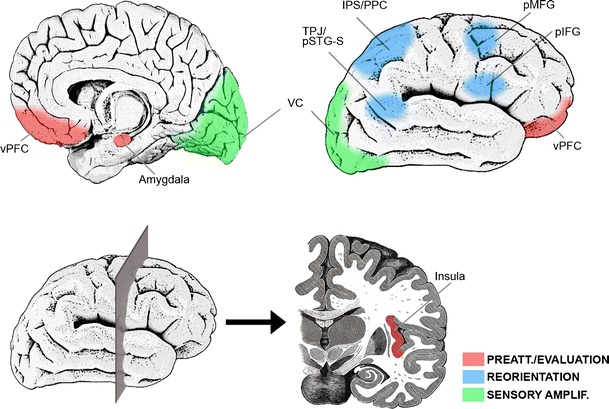
Graphical schematic summary of main spatial results reported up to date regarding exogenous attention to emotional distractors in concurrent but distinct target–distractor tasks. Organization in different subprocesses (colors) is theoretically based and, therefore, hypothetical. vPFC = ventral prefrontal cortex, VC = visual cortex, TPJ = temporo-parietal junction, pSTG-S = posterior part of the superior temporal gyrus-sulcus, IPS = intraparietal sulcus, PPC = posterior parietal cortex, pMFG = posterior middle frontal gyrus, pIFG = posterior inferior frontal gyrus

Along with temporal information, spatial data are also essential to defining cerebral mechanisms underlying exogenous attention to emotional stimuli. In order to organize available information, this section will follow the functional subprocesses previously mentioned as involved in exogenous attention: preattention/evaluation, reorienting, and sensory amplification (see the Characterization of Exogenous Attention to Nonemotional Stimuli section). Figure 6 summarizes the information presented in the following sections.

#### Preattention/evaluation network

As has been indicated, preattention consists of the low-cost, online, and fast evaluation of our environment, which works on low-level stimulus features in order to detect relevant stimulation and to trigger reorienting mechanisms (Graham, 1997; Öhman, 1979; Öhman, Flykt, & Esteves, 2001; Theeuwes, 1992). Up to now, models of exogenous attention have not clearly attributed this function to any particular node of the DAN and VAN networks, but some clues suggest that it lies, at least partially, outside them. Taking into account the functional characteristics of preattention/evaluation and the inputs and outputs proposed for this subprocess (see the Characterization of Exogenous Attention to Nonemotional Stimuli section), neural circuitry subtending it should meet several criteria. First, it should act rapidly enough to modulate other processing areas, such as the visual cortex, when the distractor is considered worth attending to; as was explained in the Temporal Characterization: Main Phases section, some type of preattention/initial evaluation should have been initiated prior to 90–100 ms, when the first discrimination between emotional and nonemotional stimulation appears to occur in visual cortices. Second, and related to the previous point, preattention/evaluation structures should receive direct sensory inputs from sensory cortices or sensory nuclei, so speed is guaranteed. Third, since preattention not only is in charge of evaluating the saliency of any unattended element within the visual scene, but also triggers attention capture processes when necessary, preattention/evaluation structures should have direct efferences to areas in charge of reorienting processing resources to the distractor (i.e., areas mainly, but not exclusively, belonging to DAN) and of enhancing sensory processing (visual cortices, in the case of visual exogenous attention). Fourth, preattention/evaluation circuitry must be relatively insensitive to processing load, since, by definition, this does not affect preattention. Fifth, it should be efficient also in magnocellular-biased conditions (see the “Magnocellular–Parvocellular Balance”: Motion, Spatial Frequency, Eccentricity section). And sixth, this circuitry must demonstrate special sensitivity to emotionally/biologically salient stimulation.

These criteria have not been systematically explored up to the moment for any brain structure in relation to exogenous attention, but existing indirect data point to several candidates: the amygdala, the ventral prefrontal cortex (vPFC), and the insula. These structures are well known to be sensitive to emotional stimuli (see reviews in Bartra, McGuire & Kable, 2013; Carretié, Albert, et al., 2009; Lindquist, Wager, Kober, Bliss-Moreau, & Barrett, 2012; Sabatinelli, Fortune, Li, Siddiqui et al., 2011). The extent to which the rest of the conditions are met by these structures is discussed next. It should be pointed out that current information does not yet support their compliance with all conditions, mainly due to the scarcity of research in some fields. For example, the systematic study of response latencies in different areas of the human vPFC, insula, or amygdala is still almost nonexistent, for obvious reasons related to the invasiveness of direct recording techniques.

The proposal here is that these structures, and other candidates that future research may reveal, form a circuit labeled here as the preattention/evaluation network (PEN), without a conspicuous core or central role for any of them, so they may modulate or complement one another’s activity. Indeed, PEN nodes are deeply interconnected (see, e.g., Emery & Amaral, 2000, for amygdala-vPFC mutual projections; Clascá, Llamas & Reinoso-Suárez, 1997, for those between the anterior insula and amygdala; and Cavada, Compañy, Tejedor, Cruz-Rizzolo, & Reinoso-Suárez, 2000, and Morecraft, Geula & Mesulam, 1992, for vPFC and insula interconnections), so that they may actually form a functional network. Importantly, PEN structures remain intact in patients in which attention, but not preattention, is affected, their lesions involving other relevant networks such as DAN (e.g., Tamietto, Geminiani, Genero & de Gelder, 2007).

##### Amygdala

The amygdala is, by far, the most widely studied structure in experiments using CDTD tasks. All studies recording fMRI during these tasks (*n* = 14), except one, predefine a region of interest (ROI) in the amygdala (Table 1). Other candidates for integrating the PEN have received much less attention up to now. This marked interest in the amygdala is well justified. Animal studies reveal the central role of the amygdala in emotional reactions and, particularly, in the urgent components, thanks to a short thalamo-amygdalar circuit (together with the long thalamo-cortical-amygdalar circuit), which permits the organism to react rapidly to danger (LeDoux, 2000). Some studies posit a direct pulvino-amygdalar transmission line, without the intervention of the visual cortex, in humans (de Gelder, Vroomen, Pourtois & Weiskrantz, 1999; Morris, Öhman & Dolan, 1999), but there is no direct anatomical evidence supporting the rapid visual subcortical thalamo-amygdalar route in our species up to date (Adolphs, 2008; Vuilleumier, 2005). The amygdala is connected to autonomic and motor executive structures, such as the hypothalamus and periaqueductal gray (PAG) area (Emery & Amaral, 2000; LeDoux, 2000), and is also capable of modulating the activity of sensory areas through its projections to auditory and visual cortices (Adolphs, 2004; LeDoux, 2000; Vuilleumier, 2005). Another criterion mentioned for preattentional structures is their capability to respond to magnocellular-biased stimuli such as those presented in the periphery. This is borne out by several studies, as is shown in Table 1 (Hodsoll et al., 2011; Hsu & Pessoa, 2007; Silvert et al., 2007; Vuilleumier et al., 2001). Moreover, the amygdala shows significant responses to low spatial frequencies within emotional stimuli (Vuilleumier et al., 2003).

While the crucial role of the human amygdala in organizing the response to emotional stimuli and in modulating attentional resources to them is consistently supported (see reviews in Adolphs & Spezio, 2007; Phelps, 2005; Pourtois et al., 2012; Vuilleumier, 2005; Wager, Phan, Liberzon & Taylor, 2003), its mandatory or central involvement (or at least, its higher hierarchy) in preattentional evaluation of the environment is currently under debate. This debate is mainly fed by studies carried out to date employing deep electrodes to electrophysiologically record the amygdala’s response latencies. Current—still scarce—data do not fit well with the proposal of rapid visual processing, since latencies surpass 140 ms (Krolak-Salmon, Hénaff, Vighetto, Bertrand, & Mauguière, 2004; Mormann, Kornblith, Quiroga, Kraskov et al., 2008; Oya, 2002; Pourtois, Spinelli, Seeck & Vuilleumier, 2010; Willenbockel, 2012). Additionally, the amygdala has been reported to be highly sensitive to processing load, which should not affect preattention (see a discussion on this issue in the Attentional Load in the Ongoing Task section). Finally, recent data show that emotional stimuli are still capable of automatically capturing attention in patients presenting amygdala lesions (Piech, McHugo, Smith, Dukic et al., 2011; but note that these lesions were unilateral). As is shown in Table 1, the majority of CDTD studies exploring the amygdala have found enhanced responses to emotional distractors (Anderson, Christoff, Panitz, De Rosa, & Gabrieli, 2003; Bishop et al., 2004; Bishop et al., 2007; Hsu & Pessoa, 2007; Lim et al., 2008; Silvert et al., 2007; Straube et al., 2006; Vuilleumier et al., 2001; see also Pourtois et al., 2010, using intracranial recordings), but there are data showing no emotion-dependent activation of the amygdala in these tasks (even in low-difficulty tasks—accuracy > 90 %; Alpers, 2009; Mitchell et al., 2007). In any case, although the core, *central* role of the human amygdala in preattention and initial evaluation is debatable according to information available to date, it seems likely that its involvement in the PEN along with other structures that complement its function.

##### Ventral prefrontal cortex

The number of fMRI studies locating ROIs in the ventral areas of the vPFC is much lower than in the case of the amygdala, but several lines of evidence suggest this as a good candidate to participate in the PEN, since it meets criteria mentioned above as necessary to belong to this network. Thus, studies on visual recognition propose the ventral (and also polar, in several experiments) prefrontal cortex (namely, Brodmann areas [BAs] 10 and 11) as a region capable of continuously monitoring the environment and of modulating, in a top-down fashion, the activity of the visual cortex (Bar, 2003; Bar, Kassam, Ghuman, Boshyan, Schmid & Dale, 2006; Kveraga, Boshyan & Bar, 2007). It both receives direct sensory inputs from early stages of the visual processing pathway (e.g., Bar et al.., 2006; Bullier, 2001; Pessoa & Adolphs, 2010) and is able to top-down regulate visual processing through its projections to the parietal and visual cortices (Bar, 2003; Bar et al., 2006; Sarter, Givens & Bruno, 2001). Along with these outputs to sensory cortices, the vPFC sends projections to areas organizing autonomic and motor response areas, such as the hypothalamus, PAG, striatum, and motor cortices (Cavada, Compañy, Tejedor, Cruz-Rizzolo et al., 2000; Ongür & Price, 2000).

Another criterion met by the vPFC for being an element of the PEN is its capacity to react to peripheral distractors: It shows greater activity in response to emotional than to neutral distractors even when stimuli appear in the far periphery (outside the parafoveal area: >10º; Carretié, Albert, et al., 2013; Carretié et al., 2005). This is in line with information suggesting that the visual information that reaches the vPFC is of a magnocellular nature but sufficient for the development of rapid evaluation processes (Bar, 2003; Bar et al., 2006; Kveraga et al., 2007).

As regards speed of response, the vPFC shows rapid responses to visual stimuli in animal studies. In nonhuman primates, vPFC responses have been recorded at 80 ms (Lamme, 2000). Deep electrode recordings in humans are still very scarce, but differential activity in the vPFC to emotional stimuli has been reported at 120 ms in response to both facial expressions and visual scenes (Adolphs, Kawasaki, Oya & Howard, 2006). Further research is necessary to extract solid conclusions on vPFC latency of response. Finally, with respect to vPFC immunity to resource engagement in the ongoing task, only two studies analyzing this cortical area have compared different levels of difficulty. Mitchell and colleagues (2007) observed greater activity in the vPFC in response to emotional distractors during the difficult task. On the other hand, Bishop and co-workers (2007) found differential vPFC emotional > neutral activity only in the low-load condition. Therefore, the only two CDTD studies exploring this issue are contradictory.

##### Insula

The insula has also been scarcely studied with respect to exogenous attention to affective stimuli, despite the evaluative role proposed for this structure (Berntson, 2011; Carretié, Albert, et al., 2009). At the same time, its capability to automatically respond to emotional information has been reported even for unconsciously perceived stimuli (Sabatini, 2009; Willenbockel, 2012). Data available up to date suggests that this frontal area meets several criteria listed above as being necessary for a structure to belong to the PEN. First, it receives direct inputs from the thalamus (mediodorsal nuclei, ventromedial nuclei, and pulvinar; Clascá et al., 1997; Critchley, 2005; Shi & Cassell, 1998) and from sensory cortices, mainly olfactory and gustatory, but also from the visual cortex (Gallese, Keysers & Rizzolatti, 2004). Moreover, the insular cortex sends outputs to response execution and sensory systems once the situation has been evaluated, such as the motor cortex (Simonyan & Jürgens, 2005), the basal ganglia (Calder, Keane, Manes, Antoun, & Young, 2000), and the PAG (Critchley, 2005), as well as to the visual cortex (Rodman & Consuelos, 1994).

As regards the capacity of the insular cortex to respond to magnocellular-biased stimuli (e.g., peripheral or perceptually degraded), no data (either positive or negative) have yet been reported in relation to exogenous attentional capture: As is shown in Table 1, distractors were always presented at fixation. However, there are data showing its capability to respond to stimuli presenting only low spatial frequencies, or even presented under the consciousness threshold (Willenbockel, 2012), suggesting a privileged processing of magnocellular information for this structure. Finally, data available up to the present on the latency of emotion-sensitive insular responses range from 140 to 300 ms (Krolak-Salmon, 2003; Ponz, Montant, Liegeois-Chauvel, Silva et al., 2014; Willenbockel, 2012), although additional research is needed in this scarcely explored field.

Only three studies using CDTD tasks located an ROI in the insula. Two of them found greater insular activation in response to emotional distractors than in response to neutral ones (Alpers, 2009; Anderson, Christoff, Panitz, De Rosa et al., 2003). The third study found enhanced insular activity to affective information, but only when endogenous attention was directed toward emotional stimuli (Straube et al., 2006). No information exists on its sensitivity to the level of difficulty in the main task.

The insula is the only one of the three PEN structures proposed here that is already included in the traditional models of exogenous attention. Specifically, it has been proposed as belonging to the VAN (e.g., see the review by Corbetta et al., 2008). Whereas this proposal reinforces the idea that the insula plays a key role in exogenous attention, the question of whether it belongs to the VAN or to the PEN (or both) requires further research, ideally involving temporally agile neural signals.

#### Reorienting: DAN and VAN

As was indicated in the Characterizations of Exogenous Attention to Nonemotional Stimuli section, neural mechanisms involved in the reorientation of gaze, head, or even body are crucial in exogenous attention. Indeed, as was also mentioned in that section, one of the main brain circuits traditionally described as underlying exogenous attention, the DAN, engages several superior parietal and dorsal frontal areas that are critical for organizing and controlling eye movements, as well as body reorientation, such as the frontal eye fields, parietal eye fields, and close areas within the superior parietal lobule, and motor and premotor cortices within the dorsal-caudal frontal cortex (see reviews in Corbetta et al., 2008; Pierrot-Deseilligny, Milea & Müri, 2004; Posner et al., 2007; D. T. Smith & Schenk, 2012). Importantly, DAN areas related to motor planning and execution are more clearly linked to exogenous than to endogenous attention, according to recent proposals (D. T. Smith & Schenk, 2012). These areas associated with motor reorienting are recruited even in covert attention tasks (i.e., those in which attention, but not gaze, must be directed to the peripheral stimulus; Grosbras, Laird & Paus, 2005), which are common in the experimental designs applied in exogenous attention studies, such as CDTD.

Due to its reorienting-related role, the DAN is expected to react to peripheral distractors. This is indeed the case: Peripheral distractors cause greater activation of the DAN than do central distractors when both are presented in the same study (Carretié, Albert, et al., 2013). Importantly, main nodes of DAN may receive visual information from early visual areas or directly from the thalamus, which may explain their extremely fast response capability (<80 ms ) (frontal eye field, Kirchner et al., 2009; superior parietal lobule, Pessoa & Adolphs, 2010). Therefore, DAN activity appears to occur, at least in part, in parallel to other exogenous attention subprocesses described in this section.

DAN function is sensitive to stimulus priority (Bisley & Goldberg, 2010; Gottlieb, 2007; Ptak, 2012; Theeuwes, 2010). Emotional stimuli, by definition important for the individual, may be conceptualized as high-priority stimuli. As is shown in Table 1, several CDTD studies have found enhanced DAN activity in response to emotional distractors, as compared with neutral ones (Bishop et al., 2007, “dorsolateral prefrontal cortex”–concretely middle frontal gyrus-; Lim et al., 2008, superior parietal lobule and middle frontal gyrus ; Carretié, Kessel, et al., 2013, precentral gyrus, BA6; Carretié et al., 2012, intraparietal sulcus and middle frontal gyrus; Schönwald & Müller, 2014, angular gyrus).

Although, so far, the role of VAN main nodes has not been so precisely drawn as in the case of DAN, it has also been associated with reorienting (e.g., Corbetta et al., 2008). As can be observed in Table 1, the activity of several VAN areas is enhanced in response to emotional distractors, as compared with neutral ones (Bishop et al., 2007, superior temporal sulcus; Carretié et al., 2005, superior temporal gyrus).

#### Sensory amplification

As has been indicated, the three elements of the PEN send back projections to the visual cortex: the amygdala, vPFC, and insula. Probably for these reasons, emotional modulation of visual perception has been reported. For example, sensitivity for perceiving the luminance contrast of a stimulus is enhanced when an emotional cue is presented previously (Phelps, Ling & Carrasco, 2006). However, this modulation does not consist of a general improvement of perception. Emotional information seems to improve the perception of magnocellular-balanced visual parameters to the detriment of parvocellular-balanced parameters. For example, Bocanegra and Zeelenberg (2011) demonstrated that emotional facial expressions enhanced rapid but coarse processing of subsequent stimuli, while reducing slower but more fine-detail processing of the visual stimulus.

Enhanced responses in the visual cortex to emotional visual stimuli, as compared with neutral stimuli, have been often and consistently reported (see reviews in Carretié, Albert, et al., 2009; Pourtois et al., 2012). As regards CDTD studies (Table 1), the enhanced activation of visual cortices is a recurring result when a whole-brain strategy of analysis is adopted (or when ROIs are defined for those areas). These effects are observed in the occipital lobe (or “cuneus”; Alpers, 2009; Carretié et al., 2004; Carretié et al., 2011; Mitchell et al., 2007; Schönwald & Müller, 2014), but also in secondary visual cortices at both the temporal lobes (fusiform gyrus in the case of faces; Carretié, Kessel, et al., 2013; Lim et al., 2008) and the parietal lobes (precuneus; Carretié et al., 2005). As was explained in the Characterization of Exogenous Attention to Nonemotional Stimuli and in the Temporal Characterization: Main Phases sections, in which the temporal dynamics of exogenous attention were described, this sensory enhancement occurs in parallel with at least part of the preattention/evaluation and reorienting processes and is visible from 90 ms to latencies beyond 500 ms. This sustained sensory amplification is probably the result of recurrent, loop mechanisms involving the rest of parallel processes characterizing exogenous attention.

### Conclusions and future directions

1. Neural indices of exogenous attention are often reported to occur early, within the first 200 ms from stimulus onset, with some reports indicating differences at 100 ms, approximately [24].
2. P2a and N2x are those ERP components most frequently showing themselves as sensitive to attentional capture by emotional distractors in CDTD tasks [24].
3. Latencies and amplitudes suggest, at least partially, parallel, rather than purely serial, processes: While preattention/evaluation is active, sensory amplification and reorienting of attention may both be active also [24].
4. Structures proposed as belonging to the PEN—the amygdala, vPFC, and insula—show enhanced responses to emotional distractors in CDTD tasks [14].
5. The VAN and, more conspicuously, the DAN are also active in CDTD tasks and show increased activity in response to emotional distractors [6].
6. Visual cortex activity elicited by emotional distractors is also greater than that produced by neutral distractors [8].

#### Future directions

Linking temporal and spatial information is necessary to functionally interpret both levels of neural information. In general, whole-brain strategies are very necessary to explore areas other than those usually focused on by ROI strategies. Particularly, further exploring the architecture of the PEN and the role of some of its nodes proposed here (namely, the vPFC and insula, very scarcely explored in this field) and other cortical and subcortical candidates is probably one of the most important future directions within the study of exogenous attention to emotional stimuli.

## Global conclusions and integration

This review leads to two main general conclusions. First, a quantitative distinction can be made as regards exogenous attention to emotional stimuli: Behavioral and neural indices of attentional capture by emotional distractors are of significantly greater magnitude than those associated with neutral distractors. However, this quantitative distinction appears to be modulated by several factors, such as individual characteristics (e.g., unspecific anxiety, which enhances exogenous attention to emotional stimuli), the affective nature of the distractor (valence, arousal level, and specific emotional contents can increase the ability to capture attention), and perhaps also the cognitive nature of the ongoing task (perceptual CDTD tasks may be more susceptible than others to interference from emotional distractors, regardless of their difficulty). The data reviewed here suggest that involvement/cognitive load in the ongoing task is not a crucial factor in explaining negative results (i.e., nonsignificant differences between exogenous attention to emotional and to neutral distractors) at the behavioral level, although it does appear to influence the response of certain brain structures, such as the amygdala.

**Fig. 7:**
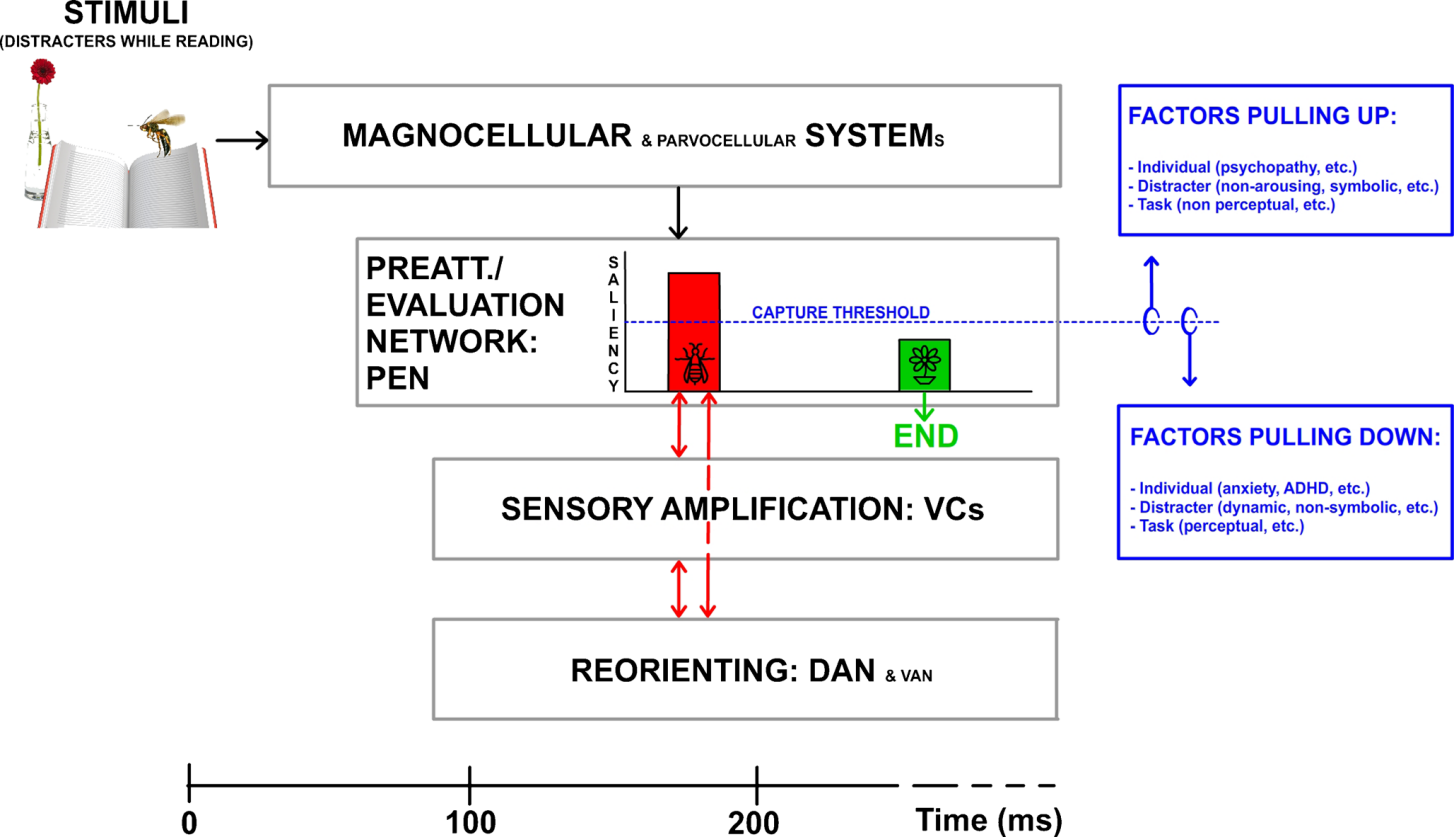
A tentative model of exogenous attention including latencies, processes, and structures revealed by research reviewed here. Two distractors are present in this illustration while the subject is reading a book: a wasp and a flower vase. The former, but not the latter, reaches the attention capture threshold during the preattention/evaluation subprocess, triggering the rest of the subprocesses. PEN = preattention/evaluation network, VCs = visual cortices, DAN = dorsal attention network, VAN = ventral attention network

Second, qualitative information on the subjacent cerebral mechanisms is also yielded by the data reviewed here. Exogenous attention to emotional stimuli reveals the involvement of neural regions that have not been described for nonemotional distractors, along with well-known mechanisms already reported. Figure 7 shows an integrated model of exogenous attention taking into account all this information. Specifically, structures related to preattention/evaluation mechanisms—widely proposed as key subprocesses in exogenous attention—such as the amygdala, vPFC, and insula, which are considered here to form the PEN, are usually left out of traditional models. Other networks and structures already identified as being involved in exogenous attention (concretely in reorienting and sensory amplification, two additional main subprocesses), such as DAN, VAN, and sensory cortices, also take part in the cerebral response to emotional distractors. However, a quantitative difference is also appreciated in this case: Stronger responses are observed in these circuits in response to emotional stimuli. In temporal terms, sensory enhancement in response to emotional distractors occurs largely in parallel with other preattentional and reorienting subprocesses and is observed from 90 to 500 ms, approximately (Fig. 7). The reviewed data suggest that exogenous attention to emotional distractors may strongly rely on the magnocellular system, an economic pathway for visual transmission and processing within the brain. Indeed, magnocellular-balanced attributes in the distractor, such as motion, low spatial frequency, or eccentricity, appear to contribute significantly to attentional capture.

From an evolutionary point of view, exogenous attention is an especially valuable tool, since continuous, low-cost monitoring of the environment and rapid reorientation to salient/emotional events are essential for survival. Several proposals for future research in this important field have been mentioned for particular contexts throughout the review. A final, more theoretical issue can be mentioned here as worthy of exploration: the single versus dual nature of exogenous attention. In the first case, exogenous attention would involve a single set of mechanisms more intensely activated by certain stimuli, including emotional stimuli; in the second, it would consist of a dual process with mechanisms that are (at least partially) distinct for neutral and emotional stimuli. Current data are still insufficient to clearly tip the balance in either direction. In any case, the segregation of “cognition” and “emotion,” although perhaps useful in some contexts, seems an inappropriate approach with regard to several important aspects of exogenous attention, and the future study of this process would be enriched through a combination of the affective and cognitive perspectives.
